# Clarifying the Use of Benzylidene Protecting Group for D-(+)-Ribono-1,4-Lactone, an Essential Building Block in the Synthesis of *C*-Nucleosides

**DOI:** 10.3390/molecules26216447

**Published:** 2021-10-26

**Authors:** Silvana Casati, Paola Rota, Pietro Allevi, Alessandra Mingione, Roberta Ottria, Pierangela Ciuffreda

**Affiliations:** 1 Dipartimento di Scienze Biomediche e Cliniche “Luigi Sacco”, Università degli Studi di Milano, Via G.B. Grassi 74, 20157 Milano, Italy; silvana.casati@unimi.it (S.C.); alessandra.mingione@unimi.it (A.M.); pierangela.ciuffreda@unimi.it (P.C.); 2 Dipartimento di Scienze Biomediche, Chirurgiche e Odontoiatriche, Università degli Studi di Milano, Via Saldini 50, 20133 Milano, Italy; paola.rota@unimi.it (P.R.); pietro.allevi@unimi.it (P.A.)

**Keywords:** ribono-1,4-lactone, 1-C-substituted nucleosides, protecting groups, cyclic acetals

## Abstract

In the last two years, nucleosides analogues, a class of well-established bioactive compounds, have been the subject of renewed interest from the scientific community thanks to their antiviral activity. The COVID-19 global pandemic, indeed, spread light on the antiviral drug Remdesivir, an adenine *C*-nucleoside analogue. This new attention of the medical community on Remdesivir prompts the medicinal chemists to investigate once again *C*-nucleosides. One of the essential building blocks to synthetize these compounds is the D-(+)-ribono-1,4-lactone, but some mechanistic aspects linked to the use of different carbohydrate protecting groups remain unclear. Here, we present our investigations on the use of benzylidene as a ribonolactone protecting group useful in the synthesis of *C*-purine nucleosides analogues. A detailed 1D and 2D NMR structural study of the obtained compounds under different reaction conditions is presented. In addition, a molecular modeling study at the B3LYP/6-31G* level of theory with the SM8 solvation model for CHCl_3_ and DMSO to support the obtained results is used. This study allows for clarifying mechanistic aspects as the side reactions and structural rearrangements liked to the use of the benzylidene protecting group.

## 1. Introduction

Nucleosides play key roles in biological processes such as the preservation, replication, and transcription of genetic information, energy storage, transmission signaling, and metabolism regulation. Nucleoside analogues can be used as nucleic acid metabolism inhibitors to interfere with viral replication and cancer cell growth, showing remarkable antiviral and antitumor effects. Consequently, the synthesis of new molecules of this class with original substitution patterns remains a challenge for medicinal chemists [1,2,3].

**Figure 1:**
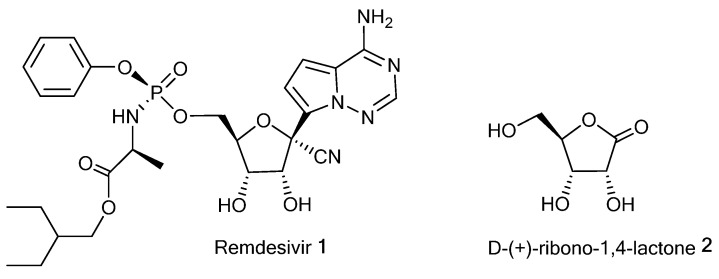
Chemical structures of the antiviral drug Remdesivir **1** and the D-(+)-ribono-1,4-lactone **2** building block.

In the last year, the Food and Drug Administration (FDA) issued an Emergency Use Authorization (EUA) for Remdesivir (**1**, Figure 1), a 1′-cyano-substituted adenine *C*-nucleoside ribose analogue, to the treatment of Corona Virus Disease 2019 (COVID-19) patients, so nucleoside-derived drugs have once again received widespread attention in the scientific community [4,5,6,7,8].

While natural and synthetic *N*-nucleosides are vulnerable to enzymatic and acid-catalyzed hydrolysis of the nucleosidic bond, their *C*-analogues are much more stable [9]. A key challenge in the preparation of this family of compounds is the coupling of the ribose with the base moieties. The different syntheses of Remdesivir (**1**) analogues reported in literature [10,11,12] involve the use of D-(+)-ribono-1,4-lactone (**2**).

Lactone **2** is a natural compound present in the leaves of *Listea japonica* and, therefore, is commercially available from renewable sources. Because of its high functionalization with contiguous chiral centers, it has been widely used as a versatile chiral building block for the construction of a variety of natural products [13,14] as well as compounds relevant to medicinal chemistry and/or chemical biology, including nucleotides and *C*-ribonucleosides as antiviral agents [15,16].

**Scheme 1:**
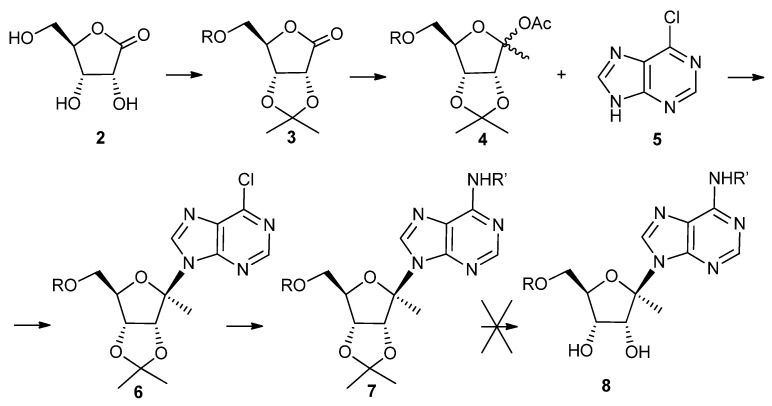
Planned pathway to achieve the 1’*C*-substituted nucleosides analogue **8**.

The appropriate protection and deprotection of the three hydroxyl groups of the D-(+)-ribono-1,4-lactone (**2**) is a fundamental aspect to be considered in the synthesis of nucleoside derivatives. Not always does the selection of a versatile and smart protecting group lead to an optimized synthetic route, sometimes it can indeed affect the result of the whole synthetic procedure, as demonstrated by our past studies [17,18]. In the synthesis of 1’-*C*-methyl-adenosines, indeed, the D-(+)-ribono-1,4-lactone (**2**) was selected as starting material to obtain compound **4**, via isopropylidene protected intermediate **3**. Then, the formation of the *N*-glicosidic bond between **4** and chloropurine **5** gave compound **6**, from which differently substituted *C*-nucleosides **7** were synthesized (Scheme 1). All attempts of deprotection, to obtain the corresponding **8**, using several of the different methods reported in the literature [19], resulted in *N*-ribosidic bond cleavage.

The use of isopropylidene as selective protection of the two neighboring hydroxyl groups in the ribonolactone **2** allows for the protection and functionalization at 5 position, fundamental for medicinal chemists to obtain libraries of potentially active nucleoside analogues. Unfortunately, it is limited to the synthesis of pyrimidine analogues, as in the purine series the acetonide deprotection is unsuccessful, probably due to the greater sensitivity of these substrates to the required acidic conditions.

The overcoming of this severe drawback remains a key goal for medicinal chemistry of purine derivatives, especially for adenosine analogues that are a relevant family of compounds with important biological and pharmacological applications [20,21,22,23]. An alternative route for the protection of the two neighboring hydroxyl groups can be the use of the benzylidene instead of the isopropylidene protecting group. Even in this case, however, unexpected reactions or structure rearrangements of the ring can occur, leading to confusing outcomes.

In this study, we report our investigation on the different reactivity of ribonolactone **2** toward either benzaldehyde or benzaldehyde dimethyl acetal in the presence of protic or Lewis acids as catalysts. The detailed study of chemical structures of the isolated compounds was performed by spectroscopic analyses, by 1D and 2D-NMR, and allowed to clarify some aspects of the use of this protecting group.

## 2. Results and Discussion

### 2.1. Syntheses and NMR Studies

Cyclic acetals and ketals [19], the most well-established and frequently used protective groups in carbohydrate chemistry synthetic strategies, have been thoroughly reviewed [24,25]. Although there are many studies published on this subject [24,26,27,28], it is not always possible to predict which isomer will be obtained under acetalization conditions. To understand and rationalize some observed results, including ring rearrangements, a detailed investigation of the reactions of D-(+)-ribono-1,4-lactone (**2**) with benzaldehyde or benzaldehyde dimethyl acetal under different reaction conditions was carried out.

**Scheme 2:**
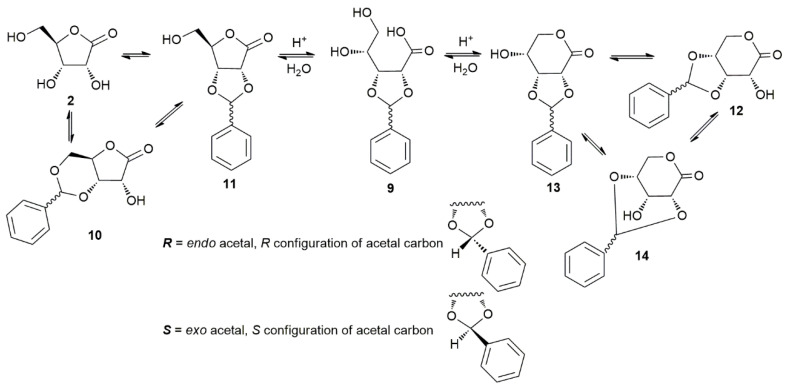
Possible products in the equilibrium mixture of the acetalization of ribonolactone **2**: two ribofuranoside acetals (**10** and **11**) and three ribopyranoside acetals (**12**–**14**) as epimeric couples at the acetalic carbon.

If we consider the studies of Han et al. [29,30], the benzylidenation of ribonolactone **2** in aqueous and thermodynamically controlled conditions can lead to five possible types of lactones passing through the ring-opening in compound **9**, the 3,5- and 2,3-benzylidene-1,4-lactones **10** and **11**, respectively, the 3,4- 2,3- and 2,4-benzylidene-1,5-lactones **12**, **13** and **14**, respectively, each of which can exist as a pair of acetal epimers (Scheme 2).

Depending on the substrate, the carbonyl compound used, and different reaction conditions, it is often possible to obtain only some of the many possible isomeric products due to the ring expansion and/or rearrangements involving acetal migration [31]. Moreover, the structural similarity of compounds **10**–**12**, combined with their easy interconversion, has led to several mistakes into structures and NMR signals assignments.

For instance, the 3,5-*O*-benzylidene structure **10** was suggested by Zinner et al. [32] in preference to the 2,3-acetal derivative **12**, for the product obtained from the reaction of D-(+)-ribono-1,4-lactone (**2**) with benzaldehyde in acidic conditions.

The correct structure was afterwards unequivocally established as 3,4-*O*-benzylidene-D-(+)-ribono-1,5-lactone (**12**) by X-ray diffraction analysis of its *O*-acetyl derivative [33]. Furthermore, 3,5-acetal derivative **10** structure was excluded [33], since no other genuine 3,5-cyclic acetals of furanoid derivatives of ribose, formed under equilibrating conditions, have been described. Moreover, molecular models suggested to the authors that a 3,5-acetal such as **10** would be more highly strained than the corresponding 2,3-acetal **11**.

In our studies on the synthetic transformations of nucleosides, the synthesis of selectively acylated acetals of D-(+)-ribono-1,4-lactone (**2**) was required. Based on the above and keeping in mind that because of equilibrium reactions the products could be of various nature, a detailed NMR investigation on the structures of the compounds obtained by the benzylidene protection of 1,4-lactone **2** under different conditions was performed and a detailed description is herein reported. Indeed, the correct identification of the compounds obtained from acetalization, reached only by an adequate NMR study and structural assignment of the spectra, is of fundamental importance to ensure the correct furanosidic structure of the ribose moiety in the final products.

**Scheme 3:**
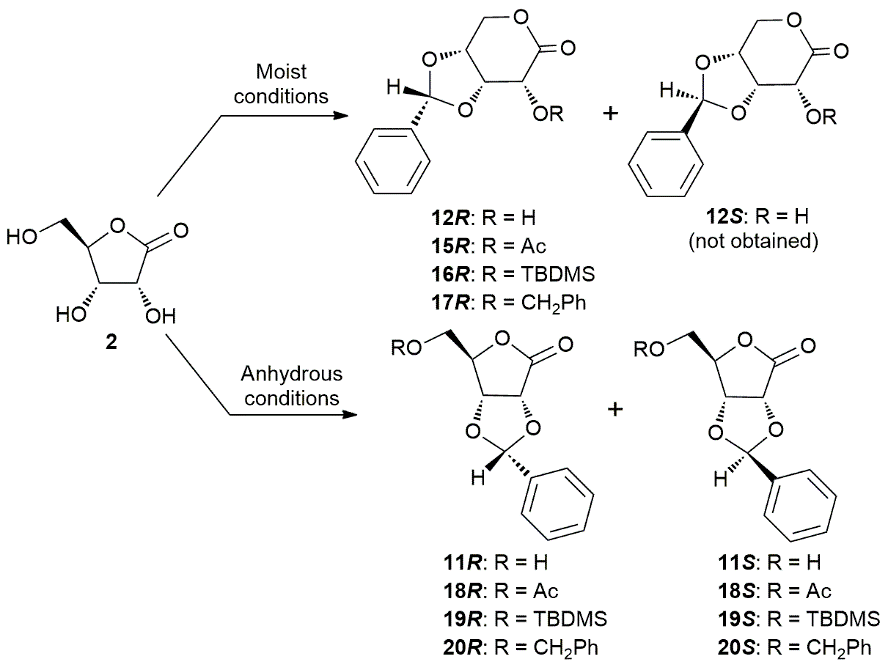
Products of benzylidenation in thermodynamic controlled moist and anhydrous conditions and corresponding derivatives.

Treatment of D-(+)-ribono-l,4-lactone (**2**) with benzaldehyde and aqueous concentrated HCl at room temperature overnight afforded, after Et_2_O addition, the 3,4-*O*-(*R*)-benzylidene-D-ribono-1,4-lactone (**12*R***, *endo*-phenyl) in high yield (81%). The insolubility of **12*R*** together with its formation under equilibrating conditions likely enables its isolation as a single diastereoisomer. Otherwise, when anhydrous ZnCl_2_ was used as a catalyst in 1,2-dimethoxyethane (DME), a diastereomeric mixture of 2,3-*O*-(*R*)-benzylidene derivative **11*R*** (55% yield) and its (*S*)-isomer **11*S*** (12% yield) was isolated. Compound **11*R*** was the major product, as previously reported [33]. Treatment of **2** with benzaldehyde dimethyl acetal and a catalytic amount of anhydrous SnCl_2_ in DME yielded compounds **11*R*** (58% yield) and **11*S*** (7% yield). The formation of **12** was not observed under non-aqueous reaction conditions, according to the literature [30] (Scheme 3).

**Scheme 4:**
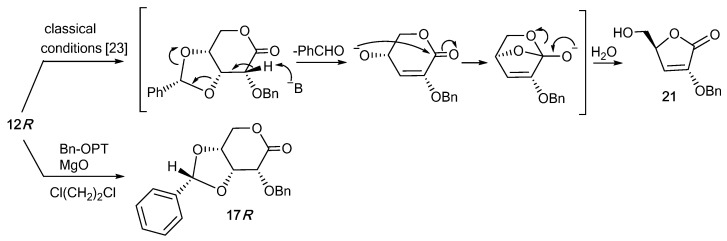
Benzylation of **12*R*** under classical conditions [20,21,22,23] and mild conditions.

The obtained compounds were additionally protected at position 5 for **11*R*** and **11*S*** or at position 2 for **12*R*** as acetate (**18*R***, **18*S***, and **15*R***), *t*-butyldimethylsilyl (**19*R***, **19*S***, and **16*R***), and benzyl (**20*R***, **20*S***, and **17*R***) groups. The acetylation and the silylation were performed using the classical reaction conditions. Also, under conventional benzylation conditions (NaH and benzyl bromide as reagents) [20,21,22,23], the compounds **11*R*** and **11*S*** afforded, as expected, the ribofuranoside benzyl derivatives **20*R*** and **20*S***, respectively. On the contrary, the same reaction procedure applied at the compound **12*R*** to synthesize the ribopyranoside **17*R*** failed. In this case, the only product isolated in good yield (69%) was the butenolide **21** (Scheme 4), deriving from an elimination-rearrangement of the pyranosidic ring.

This result was supported by Bigorra et al.’s observation on the behavior of 2-*O*-protected ribopyranoses under basic conditions [34]. In the same work [34], the authors obtained the desired ribopyranoside **17*R*** in good yields (60%), starting from compound **12*R*** and using benzyl bromide in the presence of Ag_2_O in THF. Unfortunately, by repeating the same procedure, we were not able to isolate compound **17*R***, but a mixture of unidentified substances was obtained. So, we thought to use the 2-benzyloxy-1-methylpyridinium triflate (Bn-OPT) as a benzylating agent operating under mild and nearly neutral conditions [35,36]. Under our optimal conditions, ribopyranoside **17*R*** was obtained in good yield (61%), showing correct chemical-physical properties and ^1^H and ^13^C NMR spectra in DMSO (data not reported), superimposable to those described by Bigorra et al. [34]. The different protections make these compounds available as building blocks for the various requirements of the carbohydrate chemistry strategies; moreover, they allow useful structural study.

NMR analysis techniques applied to a thorough structural study of compounds with biological or pharmacological applications is a well-established practice in medicinal chemistry [37,38,39,40] and of fundamental importance to unequivocally identify synthesis products [41]. The structural assignments of all obtained compounds (Table 1 and Table 2) were based on a detailed analysis of the 1D and 2D ^1^H- and ^13^C-NMR spectra also involving n.O.e. studies, giving significant information about all ^1^H-^1^H interactions.

^1^H-NMR studies dealing with vicinal coupling constants have revealed some useful models (Table 3) which also allow for differentiation between different sugar-related lactones. Smaller coupling occurrences between H-2 and H-3 were found for pyranoside lactones **12*R***–**17*R*** (*J*_2,3_ = 3.2–3.3 Hz) in comparison with those for furanoside lactones **11*R***, **18*R***–**20*R*** and **11*S***, **18*S***–**20*S*** (*J*_2,3_ = 5.6–6.0 Hz). In addition, high values had been found for H-3, H-4 coupling in **12*R***–**17*R*** (*J*_3,4_ = 8.1–8.4 Hz), while they are nearly absent in the *R* and *S* epimers of **11**, **18**–**20** (*J*_3,4_ < 1 Hz). The n.O.e. experiments performed with selected lactones disclosed other important information about the structural characteristic, and also confirmed the results deduced from coupling constant analysis. The correlations between H-3 and H-4 in compound **12*R*** and their absence in the *R* and *S* epimers of compound **11** suggested that, in the first compound, H-3 and H-4 are *syn*, as in the pyranoside ring, while in the second ones, the H-3 and H-4 are *anti*, as in the furanoside ring.

^13^C-NMR spectroscopy has been previously proposed as a useful tool to establish the size of the lactone ring, supported by considerable differences in the chemical shifts found for C-3 and C-4 in the lactones studied [43]. Thus, the chemical shifts of C-3 and C-4 in the six-membered lactones (**12*R***, **15*R***–**17*R***) are all within the range of 68.0–77.8 ppm (Table 2), whereas in the five-membered lactones (**11**, **18**–**20**), the C-3 resonates in the range of 77.7–81.7 ppm and C-4 resonates at lower field in the range of 79.0–84.5 ppm. Also, the signal for C-5 in the lactones **12*R***, **15*R***–**17*R*** is at a lower field (67.0–67.5 ppm) than that for C-5 in both *R* and *S* epimers of furanose lactones **11**, **18** and **19** (62.3–63.6 ppm). Only compounds **20*R*** and **20*S*** make an exception to this trend in chemical shifts due to the benzoylation at the position 5.

In addition, in previous reports [33,44], describing both acetalic epimers of the 2-phenyl-1,3-dioxolanes, the relative *R* and *S* configuration was identified by (i) the acetal proton signal which is normally found at a higher field in the *R* than in the corresponding *S* isomer and (ii) the ^13^C shift of the acetal carbon atom is found at a higher field in the *S* than in the corresponding *R* isomer, in CDCl_3_ solutions. In our case, for the compounds **11**, **18**–**20**, while ^13^C NMR results (Table 2) are consistent with the previous observations, the acetal proton signals in the ^1^H-NMR spectra are not significant to discriminate between the two epimers (Table 1).

Finally, the correct identification of the two epimers **11*R*** and **11*S*** can be unequivocally obtained by n.O.e. experiments, where only in the *R* isomer the acetal proton correlates with the H-2 and H-3 protons, due to their presence at the same side of the carbohydrate ring.

### 2.2. Computational Studies

To rationalize the described results and to obtain further useful information on the acetalization reaction described in the work, a molecular modeling study was carried out on the *R* and *S* acetal epimers of the compounds **10**, **11**, **12** and **13** (Scheme 2). Compound **14** was excluded due to the high steric strain in its structure, which makes its existence unlikely.

The conformational space of each compound was explored with a molecular mechanics model, the MMFF force field implemented in Spartan 14 (see experimental) obtaining a selection of low-energy conformers. The structures of the conformers obtained were subjected (in vacuum) to a higher-level geometry optimization and a single-point energy calculation by a hybrid DFT approach at the B3LYP/6-31G* level.

Then, based on the relative energy differences of each conformer compared to that of the corresponding global minimum, the population at 298 K of conformers for each compound, according to the Boltzmann equation, has been estimated.

Finally, the energies of the different compounds were calculated as a weight average based on the energy and the corresponding mole fraction of their conformers.

In the benzylidenation reaction carried out in the presence of an aqueous acid, all the equilibrium represented in Scheme 2 are operative.

On the basis of the energies calculated for the various potential products, the mixture obtained from this reaction, under thermodynamic control, should consist almost exclusively of the compound **12*R*** (62.5%), which has the lowest energy, accompanied by its epimer **12*S*** (36.3%, ΔE_rel_ = 1.34 kJ/mol), while the other compounds **11*R*** (ΔE_rel_ = 11.75 kJ/mol), **11*S*** (ΔE_rel_ = 12.39 kJ/mol), **13*S*** (ΔE_rel_ = 15.34 kJ/mol), **13*R*** (ΔE_rel_ = 16.41 kJ/mol), **10*R*** (ΔE_rel_ = 41.36 kJ/mol), and **10*S*** (ΔE_rel_ = 45.24 kJ/mol), should be present only in trace amounts.

In the reaction in the presence of concentrated HCl described in this work, only the compound **12*R*** in 81% yield was obtained by separation after addition of Et_2_O to the reaction mixture. The obtaining of only the compound **12*R*** with a higher yield than that expected by molecular modeling calculations could be explained both by considering that its insolubility, and therefore its separation from the mixture during the proceeding of the reaction, can shift the equilibrium towards its formation, and the possibility that its **12*S*** epimer, if present, could remain in the reaction mixture after the direct separation of **12*R*** by Et_2_O addition.

The reaction between ribonolactone **2** (and other pentonolactones) and benzaldehyde was investigated in detail in 1994 by Han et al. [29] by molecular mechanics (MM2) and semiempirical (PM3 and AM1) calculations on the conformation and thermodynamic stability of the main products, to obtain information on the equilibrium composition of the reaction.

The data obtained from these calculations, however, show that none of the computational methods utilized produced completely correct results. In fact, the molecular mechanics calculations often accurately reproduce the structural geometry of molecules but do not give reliable energy data, for different structural isomers, while semiempirical methods predict more reliable energy values, but are less accurate in predicting the geometric structure of compounds. On the other hand, in the years in which the work was done, higher-level calculations, such as ab initio approaches that give structural geometry and energy results more accuracy, still had excessive computational costs for the relatively large molecules studied.

When the benzylidenation reaction is carried out under non-aqueous conditions, the ring expansion from furanosic 1,4-lactone to give pyranosic 1,5-lactone, via intermediate aldonic acid **9** derivatives, cannot take place. In this case, only the *R* and *S* acetal epimers of compounds **10** and **11** can be formed. Based on the energies calculated for these compounds, the thermodynamic equilibrium mixture of the reaction under anhydrous conditions should consist of **11*R*** (56.5%), which has the lowest energy and its **11*S*** epimer (43.5%, ΔE_rel_ = 0.64 kJ/mol), with only traces of compounds **10*R*** (ΔE_rel_ = 29.61 kJ/mol) and **10*S*** (ΔE_rel_ = 33.49 kJ/mol).

To obtain useful information in support of the results of the NMR studies on the *R* and *S* epimers of compounds **11** and **12**, the energy of their conformers, and consequently, the distribution of the corresponding populations, was recalculated considering the solvents used in the NMR experiments. For each conformer, the energy was re-optimized at the B3LYP/6-31G* theory level with the SM8 solvation model, included in Spartan 14, to consider the solvation energy because of the solvent environment (CHCl_3_ for **11** and DMSO for **12**). Then, based on the calculated energies, the Boltzmann distribution of the conformer populations of the *R* and *S* epimers of **11** in CHCl_3_ and **12** in DMSO was determined (Tables S1–S4 in Supplementary Materials).

**Figure 2:**
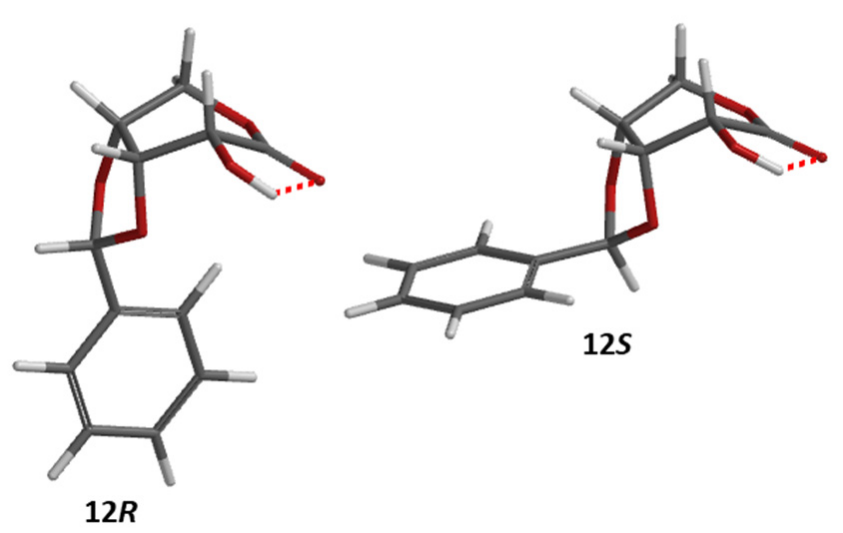
Lower energy conformer calculated in DMSO at 298 K, of **12*R*** (>99% of population) and **12*S*** (>99% of population).

The results of the calculations indicate that the compounds **12*R*** and **12*S*** in DMSO at 298 K are present for more than 99.9% in a single conformation (Figure 2, Tables S1 and S2 in Supplementary Materials). In both compounds, there is an intramolecular hydrogen bond between the hydroxyl in position 2 and the oxygen of the adjacent carbonyl with the followed geometry: for **12*R*** O···H distance = 2.084 Å, H-O distance = 0.976 Å; H···O-H angle = 117.90°, for **12*S*** the corresponding values are: 2.070 Å, 0.977 Å and 118.60°.

Furthermore, for both compounds, the 1,5 lactone ring assumes a boat conformation (^2,5^B), as described by the puckering parameters [45,46], identical for the two compounds (puckering amplitude *Q* = 0.70 Å, pseudorotation phase angles *Φ* = 86° and *ϕ* = 127°).

**Figure 3:**
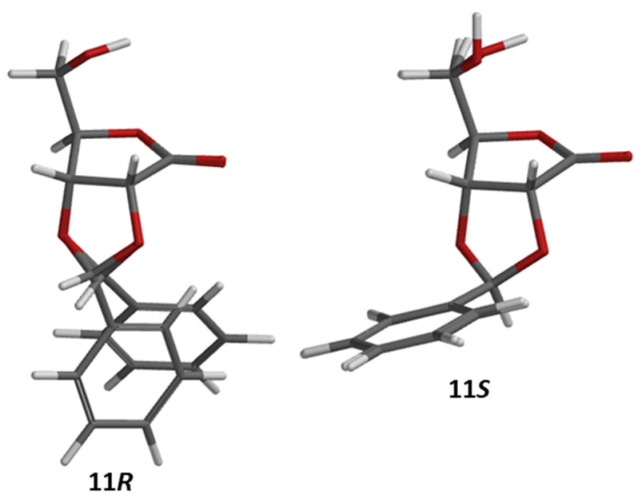
Superposition of the lactone ring of the two lower-energy conformers, calculated in CHCl_3_ at 298 K of **11*R*** (95% of the population) and **11*S*** (88% of the population).

In the case of **11**, its *R* and *S* epimers in CHCl_3_ at 298 K are present as mixtures of conformers. Compound **11*R*** consists of more than 95% of two conformers in a ratio of 89:11 (Figure 3, Table S3 in Supplementary Materials). The two conformers have the lactone ring atoms perfectly superimposable in space and the furanoside lactone ring assumes an envelope (*E*_3_) conformation as described by the puckering parameters (puckering amplitude *q*_2_ = 0.22 Å and pseudorotation phase angle *ϕ*_2_ = 108°).

Finally, the compound **11*S*** in CHCl_3_ at 298 K is present as a mixture of conformers, constituted for over 88% by two conformers in the ratio 71:29 (Figure 3, Table S4 in Supplementary Materials). These two conformers mainly differ in the different orientation of the 4-hydroxymethyl group, but also for these conformers the lactone ring atoms are perfectly superimposable in space.

The puckering parameters of the furanosidic lactone rings, identical for these two main conformers (puckering amplitude *q*_2_ = 0.20 Å and pseudorotation phase angle *ϕ*_2_ = 122°), indicate a conformation of the ring intermediate between twist and envelope (^4^*T*_3_*/E*_3_).

The values of the coupling constants of the vicinal protons for the compounds **11*R***, **11*S*** and **12*R*** were then calculated.

For each conformer present in the populations of these compounds, the vicinal ^1^H coupling constants were calculated from the corresponding H-C-C-H dihedral angles using a Karplus-type equation with the Haasnoot–Altona parameterization [42], accounting for the coupling dependence not only on the dihedral angle, but also on the electronegativity of the participating atoms, and on the orientation of the α- and β-substituents. Then, the values of the ^3^*J*(H,H) coupling constants in the compounds **11*R***, **11*S*** and **12*R*** were obtained weighting average based on the population percentages.

The calculated values (Table 3) for these constants agree with those experimentally observed, thus strongly supporting the assignments made.

In a similar way, the distances between the hydrogen atoms in the compounds (Table 4) were calculated as the weight average of the corresponding distance in the individual conformers and their abundance in the population.

## 3. Material and Methods

### 3.1. Chemistry

Chemicals and solvents were obtained from Sigma-Aldrich (Merck, Darmstadt, Germany) and used without further purification. Column chromatography was performed on silica gel 60 (70–230 mesh) using the specified eluents. The progress of the reactions was monitored by analytical thin-layer chromatography (TLC) on pre-coated glass plates (silica gel 60 F254-plate-Merck, Darmstadt, Germany) and the products were visualized by UV light, anisaldehyde/H_2_SO_4_/EtOH solution with heat as the developing agent. Optical rotations were taken on a PerkinElmer 241 polarimeter equipped with a 1 dm tube; [α]_D_ values are given in 10^−1^ deg cm^2^ g^−1^. Purity of all compounds (>99%) was verified by thin-layer chromatography and NMR measurements [47,48,49,50].

### 3.2. NMR Analysis

NMR spectra were recorded on a Bruker AVANCE 500 spectrometer equipped with a 5 mm broadband reverse probe and deuterium lock with field z-gradient operating at 500.13 and 125.76 MHz for ^1^H and ^13^C, respectively. All NMR spectra were recorded at 298 K in CDCl_3_ or in DMSO-*d*_6_ (isotopic enrichment 99.98%) solution and the chemical shifts were reported on a δ (ppm) scale. The central peak of DMSO-*d*_6_ signals (2.49 ppm for ^1^H and 39.50 ppm for ^13^C) and of CDCl_3_ signals (7.26 ppm for ^1^H and 77.0 ppm for ^13^C) were used as internal reference standard. Acquisition parameters for 1D were as follows: ^1^H spectral width of 5000 Hz and 32 K data points providing a digital resolution of ca. 0.305 Hz per point, relaxation delay 2 s; ^13^C spectral width of 29,412 Hz and 64 K data points providing a digital resolution of ca. 0.898 Hz per point, relaxation delay 2.5 s. The experimental error in the measured ^1^H-^1^H coupling constants was ± 0.5 Hz.

### 3.3. Computational Procedures

Computational study of compounds **10*R***–**13*R*** and **10*S***–**13*S*** was performed with the software Spartan ’14 (Wavefunction, Inc., Irvine, CA, USA) using the default parameters unless otherwise indicated.

Each compound was subjected to a systematic search of the conformational space using MMFF force field in vacuum, to identify the low-energy conformers, and the structures within 50 kJ/mol of the global minimum were saved. The used “conformer distribution” function in Spartan automatically utilized the systematic search method due to the low number of conformational degrees of freedom in the studied molecules.

The geometry of the obtained conformers was fully re-optimized (“Equilibrium Geometry” function) at a higher-level geometry optimization using a density functional method (B3LYP) with the 6-31G* basis set. A single-point energy calculation was then performed (“Energy” function with into options for the keywords: GRADIENTTOLERANCE = 1E-9, OPTCYCLE = 30,000 and GEOMETRYCYCLE = 30,000).

To confirm that the structures are in local minima, the normal-mode frequencies of the optimized conformational isomers were calculated. Each vibration spectrum does not show any negative frequency values, confirming that the optimized structure is at the lowest point.

The conformer populations for each compound were calculated based on the relative energy of a given conformer with respect to the global minimum, using the well-known Boltzmann distribution equation.

The energies for the compounds **10*R***–**13*R*** and **10*S***–**13*S*** were calculated as the weight average considering for each compound, the energy of the single conformers and the mole fraction with which they are present in the conformer population.

For the NMR considerations, the energies of each conformer of **11*R***, **11*S***, **12*R*** and **12*S*** were re-optimized at the B3LYP/6-31G* level using the SM8 solvation model [51] included in the Spartan program package.

In the B3LYP/6-31G* single-point energy calculation, in addition to those indicated above, the keywords POSTSOLVENT = TRICHLOROMETHANE for **11*R*** and **11*S*** and POSTSOLVENT = DIMETHYLSULFOXIDE for **12*R*** and **12*S*** were used. The energies thus obtained were utilized to determine the Boltzmann distribution of the conformers in CHCl_3_ for the compounds **11*R*** and **11*S*** in DMSO for the compounds **12*R*** and **12*S***.

The conformations of the furanose lactone rings in the main conformer of **11*R*** and of **11*S*** and those of the pyranose lactone rings in the main conformer of **12*R*** and **12*S*** were determined using Cremer–Pople puckering parameters [44], which define the conformation of a puckered ring in a quantitative and mathematically well-defined way [45].

For each conformer of the population of compounds **11*R***, **11*S*** and **12*R***, the vicinal proton–proton coupling constants, ^3^*J*(H,H), were calculated from the corresponding H-C-C-H torsion angles using the Altona equation [42] based on the Pauling electronegativity of α and β position atoms.

The ^3^*J* coupling constants and the distance between hydrogen atoms for compounds **11*R***, **11*S*** and **12*R*** were calculated as the weight average considering the corresponding value in each conformer and the mole fraction with which the conformer is present in the population.

### 3.4. 3,4-O-(R)-Benzylidene-D-Ribono-1,5 Lactone (12R)

To a solution of D-(+)-ribono-1,4-lactone **2** (1.00 g, 6.75 mmol) in benzaldehyde (9 mL), concentrated hydrochloric acid (0.9 mL) was added and the mixture was stirred at room temperature. After 20 minutes, the formation of a precipitate begins, and it increases during the time. The reaction was stirred at room temperature overnight, and then, Et_2_O (20 mL) was added to the semi-solid heterogeneous reaction mixture. A white solid was collected by filtration, and it was washed with 10% aqueous sodium bicarbonate (10 mL) and water (10 mL). Finally, the product was refluxed for 20 minutes in acetone (100 mL), then the mixture was left to cool at room temperature and the solid was filtered, yielding **12*R*** as a pure white solid (1.29 g, 81% yield), TLC (hexane/AcOEt; 3:7 *v*:*v*) R_f_ 0.25; mp 232–234 °C [lit. [30] 230–231.5 °C]; [α]^25^_D_ − 173 (c 1.0, DMF) [lit. [30] − 180.5 (c 0.47, DMF)].

### 3.5. 2,3-O-(R)-Benzylidene-D-Ribono-1,4-Lactone (11R) and 2,3-O-(S)-Benzylidene-D-Ribono-1,4-Lactone (11S)

Method A: D-(+)-ribono-1,4-lactone **2** (1.00 g, 6.75 mmol) was suspended with vigorous stirring in dry 1,2-dimethoxyethane (3 mL)-containing benzaldehyde (5 mL, 49.23 mmol), then ZnCl_2_ (0.90 g, 6.60 mmol) was added, and the mixture was stirred at room temperature under an argon atmosphere. After 5 h, water (5 mL) was added and the mixture was extracted with AcOEt (3 × 5 mL), the organic layer was washed with water and aqueous NaHCO_3_, dried over anhydrous Na_2_SO_4_ and evaporated in vacuum. The residue was purified by flash chromatography on silica gel (hexane/AcOEt 6:4 *v*:*v*) to afford the compounds **11*R*** (877 mg, 55% yield) and **11*S*** (191 mg, 12% yield) as white solids. Compound **11*R*** showed: TLC (hexane/AcOEt 7:3 *v*:*v*) R_f_ 0.13; mp 164–165 °C, [lit. [30] 156–158 °C, [34] 163–164 °C]; [α]^22^_D_ −78.0 (c 0.5, CHC1_3_) [lit. [30] −79.1 (c 0.56, CHC1_3_) −70 (DMF)]. Compound **11*S*** showed: TLC (hexane/AcOEt 7:3 *v*:*v*) R_f_ 0.21; mp 86–87 °C [lit. [31] 83–84 °C, [34] 87–88 °C]; [α]^22^_D_ −38 (c 1.0, CHC1_3_) [lit. [31] −39.3 (c 1.16, CHC1_3_), [34] −40 (CHC1_3_)].

Method B: To a suspension of lactone **2** (1.00 g, 6.75 mmol) in dry 1,2-dimethoxyethane (4 mL) were added benzaldehyde dimethyl acetal (1.32 mL, 8.79 mmol) and a catalytic amount of anhydrous SnCl_2_ (11 mg, 0.06 mmol). The reaction mixture was heated to reflux and stirred for 5 h under an argon atmosphere. After evaporation of the solvent under vacuum, the residue was purified by silica gel flash-column chromatography (hexane/AcOEt 6:4 *v*:*v*) to afford compounds **11*R*** (925 mg, 58% yield) and **11*S*** (112 mg, 7% yield) as white solids.

### 3.6. General Procedure for the Acetylation

To a solution of **11** or **12** (0.40 mmol) in pyridine (1 mL), acetic anhydride (0.06 mL, 0.63 mmol) was added, and the reaction was stirred at room temperature for 3 h. Then, the reaction mixture was diluted with CH_2_Cl_2_ (5 mL), washed with 2 M HCl (3 × 5 mL), saturated solution of NaHCO_3_ (5 mL), water (5 mL) and dried over anhydrous Na_2_SO_4_. The solvent was evaporated under reduced pressure and the crude residue was purified by flash chromatography on silica gel.

Starting from **11*R***, the acetyl derivative **18*R*** was obtained (101 mg, 91% yield) showing: TLC (hexane/AcOEt 7:3 *v*:*v*); R_f_ 0.31; mp 144–145 °C [lit. [31] 143–144 °C]; [α]^22^_D_ −51 (c 1, CHC1_3_) [lit. [31] −72.5 (c 1.48, CHC1_3_)].

Starting from **11*S***, the acetyl derivative **18*S*** was obtained (99 mg, 89% yield) showing: TLC (hexane/AcOEt 7:3 *v*:*v*); R_f_ 0.46; mp 85–86 °C (dec); [α]^22^_D_ −4 (c 1, CHC1_3_).

Starting from **12*R***, the acetyl derivative **15*R*** was obtained (103 mg, 93% yield) showing: TLC (hexane/AcOEt 1:1 *v*:*v*); R_f_ 0.26; mp 174–176 °C [lit [34] 173–174 °C]; [α]^22^_D_ −93 (c 1, CHC1_3_).

### 3.7. General Procedure for the Silylation

To a stirred solution of **11** or **12** (0.4 mmol) in CH_2_Cl_2_ (3 mL) containing trimethylamine (120 μL, 0.87 mmol) and dimethylaminopyridine (5 mg, 0.04 mmol), tert-butyldimethylsilyl chloride (125 μL, 0.72 mmol) was added. The solution was stirred overnight at room temperature and under argon atmosphere. Then, the mixture was washed in succession with 1 M HCl (2 mL), a saturated solution of NaHCO_3_ (3 mL), water (3 mL) and dried over anhydrous Na_2_SO_4_. After evaporation under reduced pressure, the residue was purified by flash chromatography on silica gel.

Starting from **12*R***, the silyl derivative **16*R*** was obtained (123 mg, 88% yield) showing: TLC (hexane/AcOEt 7:3 *v*:*v*); R_f_ 0.39; mp 120–122 °C, [α]^22^_D_ −120 (c 1, CHC1_3_) [lit. [52] −134 (c 1, CHC1_3_)].

Starting from **11*R***, the silyl derivative **19*R*** was obtained (115 mg, 82% yield) showing: TLC (hexane/AcOEt 7:3 *v*:*v*); R_f_ 0.83; mp 130–131 °C; [α]^22^_D_ −46 (c 0.5, CHC1_3_).

Starting from **11*S***, the silyl derivative **19*S*** was obtained (120 mg, 86% yield) showing: TLC (hexane/AcOEt 7:3 *v*:*v*); R_f_ 0.89; mp 92–93 °C (dec); [α]^22^_D_ +6 (c 0.5, CHC1_3_).

### 3.8. General Procedure for the Benzylation

To a stirred solution of **11** or **12** (0.4 mmol) in anhydrous THF (2 mL) cooled to 0 °C, a 60% NaH dispersion (19 mg, 0.48 mmol) was added. Benzyl bromide (57 μL, 0.48 mmol) was then added at the same temperature. The reaction mixture was allowed to warm to room temperature and then stirring was continued overnight. The mixture was then slowly poured into ice water (2 mL) and extracted with CH_2_Cl_2_ (3 × 2 mL), the organic layer was washed with water (2 × 3 mL) and dried over anhydrous Na_2_SO_4_. The solvent was evaporated under reduced pressure and the residue was purified by flash chromatography on silica gel.

Starting from **11*R***, the benzyl derivative **20*R*** was obtained as an oil (68 mg, 52% yield) showing: TLC (hexane/AcOEt 7:3 *v*:*v*); R_f_ 0.55; [α]^22^_D_ −125 (c 0.5, CHC1_3_).

Starting from **11*S***, the benzyl derivative **20*S*** was obtained as an oil (73 mg, 56% yield) showing: TLC (hexane/AcOEt 7:3 *v*:*v*); R_f_ 0.51; [α]^22^_D_ +28 (c 1, CHC1_3_).

Starting from **12*R*** in 1:1 THF/DMF mixture (2 mL), the only product formed and isolated by flash chromatography (hexane/AcOEt 3:7 *v*:*v*) was the (*S*)-3-benzyloxy-5-hydroxymethyl-2(5H)-furanone (**21**), as a white solid (61 mg, 69% yield) showing: TLC (hexane/AcOEt 3:7 *v*:*v*); R_f_ 0.37; mp 99–102 °C [lit. [35] 99–101 °C]; [α]^22^_D_ +22 (c 1, MeOH) [lit. [35] +9.4 (c 0.85, MeOH), [53] +11.4 (c 1, MeOH) ]; ^13^C NMR (CDCl_3_) 63.6, 72.9, 79.3, 114.6, 127.6, 128.6, 128.7, 134.6, 146.8, 167.4 [lit. [35] ^13^C NMR (CDCl_3_) 63.6, 73.0, 79.3, 114.8, 127.7, 128.6, 128.7, 134.8, 146.9, 167.4].

### 3.9. 2-O-Benzyl-3,4-O-(R)-Benzylidene-D-Ribono-1,5-Lactone (17R)

To a solution of compound **12*R*** (100 mg, 0.42 mmol) in dichloroethane (14 mL), 2-benzyloxy-1-methylpyridinium triflate (211 mg, 0.84 mmol) and MgO (34 mg, 0.85 mmol) were added. The reaction was refluxed for 24 h. The reaction was cooled to room temperature and filtered through Celite. The filtered element was concentered under vacuum and purified on silica gel by flash-column chromatography (hexane/AcOEt 6:4 *v*:*v*) to afford compounds **17*R*** (83 mg, 61% yield) as white solid.

Compound **17*R*** showed: TLC (AcOEt/hexane; 1:1 *v*:*v*) R_f_ 0.27; mp 172–175 °C [lit. [35] 178–180 °C]; [α]^25^_D_ −90 (c 1.0, DMF) [lit. [35] −123.1 (c 1.17, DMF)].

## 4. Conclusions

Over the past year, COVID-19 has sparked new interests in researching *C*-nucleoside analogues, such as Remdesivir (**1**). A key building block of this family of compounds is the D-(+)-ribono-1,4-lactone (**2**), which is then reacted with the base or base-like moiety, to obtain the *C*-Nucleosides. When *C*-nucleoside analogues incorporate purines or purine-like bases, the benzylidene group is the best choice to protect the hydroxyl groups in positions 2 and 3, even though some unexpected by-products often occur due to carbohydrate ring rearrangements. Here, we present a detailed structural investigation, also using DFT computational modeling calculations, on the compounds obtained when benzylidene is used as a protective group. This study allows to clarify some aspects related to the formation of by-products and rearrangements of the carbohydrate rings. The different reactivity of lactone **2** toward either benzaldehyde or benzaldehyde dimethyl acetal under different reaction conditions is associated with relatively minor changes in structure. Structural assignments for *O*-benzylidene-D-ribono-1,4-lactones were achieved using conventional NMR/n.O.e. techniques. In a general sense, the observed correlation between H-3 and H-4 is characteristic for D-ribono-1,5-lactones, but not for D-ribono-1,4-lactones. In addition, significant spin–spin interactions between H-4 and H-5 are present in D-ribono-1,4-lactones. The trends featured by D-ribono-1,4-lactones can be helpful resources to account for their physicochemical characterization in a simple and reliable way. The study herein presented, with the unequivocal identification of the NMR signals characteristics of the five-membered or six-membered carbohydrate rings, allows to identify which synthetic strategies lead to the undesired six-membered ring formation.

## Figures and Tables

**Table 1:** ^1^H NMR chemical shifts (ppm) of *R* and *S* epimers of **11**, **18**–**20** and *R* epimers of **12**, **15**–**17.**

Proton	11*R* CDCl_3_	11*S* CDCl_3_	12*R* DMSO	15*R* CDCl_3_	16*R* CDCl_3_	17*R* CDCl_3_	18*R* CDCl_3_	18*S* CDCl_3_	19*R* CDCl_3_	19*S* CDCl_3_	20*R* CDCl_3_	20*S* CDCl_3_
**2**	4.99	5.09	4.64	5.55	4.50	4.11	4.94	5.04	4.87–4.90 ^b^	5.0	4.95	5.08
**3**	4.94	4.87	4.70	4.87	4.73	4.78	4.88	4.79	4.87–4.90 ^b^	4.81	4.88	4.80
**4**	4.79	4.82	4.62	4.73	4.60	4.56	4.92	4.95	4.76	4.80	4.81	4.84
**5a**	3.89	3.89	4.33	4.40	4.23	4.15	4.31	4.30	3.88	3.88	3.72–3.81 ^a^	3.73–3.78 ^a^
**5b**	4.05	4.04	4.43	4.62	4.48	4.49	4.44	4.43	3.95	3.94		
O_2_C**H**Ph	6.00	6.00	5.74	5.83	5.79	5.76	6.03	6.02	5.99	5.98	5.98	6.03
**aromatics**	7.42–7.50 ^b^	7.41–7.49 ^b^	7.39–7.44 ^b^	7.39–7.49 ^b^	7.39–7.48 ^b^	7.29–7.50 ^b^	7.42–7.49 ^b^	7.42–7.48 ^b^	7.42–7.50 ^b^	7.40–7.43 ^b^	7.28–7.48 ^b^	7.36–7.48 ^b^
OC**H**_2_Ph						4.74 5.10					4.52 4.62	4.51 4.62
OCOC**H**_3_				2.28			2.13	2.11				
C**H**_3_Si					0.17				0.12	0.10		
C**H**_3_Si					0.25				0.11	0.11		
(C**H**_3_)_3_Si					0.98				0.91	0.91		

^a^ AB part of ABX system. ^b^ overlapped.

**Table 2:** ^13^C NMR chemical shifts (ppm) of *R* and *S* epimers of **11**, **18**–**20** and *R* epimers of **12**, **15**–**17.**

Carbon	11*R* CD_3_OD	11*S* CDCl_3_	12*R* DMSO	15*R* CDCl_3_	16*R* CDCl_3_	17*R* CDCl_3_	18*R* CDCl_3_	18*S* CDCl_3_	19*R* CDCl_3_	19*S* CDCl_3_	20*R* CDCl_3_	20*S* CDCl_3_
**1**	176.1	173.9	171.9	165.5 ^a^	169.4	168.4	169.8 ^a^	169.7 ^a^	172.9	173.0	173.3	174.6
**2**	77.3	76.4	73.1	68.4	70.0	72.9	75.5	75.3	75.9	76.4	75.9	73.4
**3**	81.7	77.7	76.1	74.5	77.8	75.9	79.5	78.8	81.6	77.8	80.2	78.8
**4**	84.2	84.5	68.0	73.4	73.5	73.4	79.0	79.3	80.1	84.1	80.5	82.8
**5**	62.4	62.3	67.0	67.5	67.3	67.2	63.6	63.5	63.0	63.2	69.1	68.9
O_2_**C**HPh	107.7	104.9	104.0	105.0	104.7	104.8	107.3	107.2	106.6	104.6	106.9	104.7
aromatics	128.1 129.4 131.1 137.5	126.6 128.7 130.0 135.8	126.9 128.1 129.9 134.7	127.3 128.6 130.4 134.5	126.6 128.5 130.2 135.4	136.7 134.8 130.2 128.7 128.5 128.3 128.3 127.4	126.9 128.7 130.4 135.0	126.8 128.6 130.2 134.9	126.8 128.5 130.0 135.3	126.6 128.5 129.8 135.7	127.0 127.9 128.3 128.7 128.8 130.2 135.4 137.0	127.3 127.9 128.1 128.4 128.8 130.1 135.0 136.8
OCO**C**H_3_				20.5			20.8	20.6				
O**C**OCH_3_				169.9 ^a^			172.3 ^a^	172.2 ^a^				
O**C**H_2_Ph						72.8					74.1	72.6
**C**H_3_Si					−4.2 −5.2				−5.6 −5.8	−5.6 −5.8		
(**C**H_3_)_3_CSi					25.9				24.9	25.8		
**C**-Si					18.6				18.2	18.2		

^a^ from HMBC.

**Table 3:** ^1^H NMR coupling constants (Hz)^a^ of *R* and *S* epimers of **11**, **18**–**20** and *R* epimers of **12**, **15**–**17.**

Coupled Protons	11*R* CDCl_3_	11*S* CDCl_3_	12*R* DMSO	15*R* CDCl_3_	16*R* CDCl_3_	17*R* CDCl_3_	18*R* CDCl_3_	18*S* CDCl_3_	19*R* CDCl_3_	19*S* CDCl_3_	20*R* CDCl_3_	20*S* CDCl_3_
	exp (calc) ^b^	exp (calc) ^b^	exp (calc) ^b^	exp	exp	exp	exp	exp	exp	exp	exp	exp
**2, 3**	6.0 ^a^ (6.96) ^b^	5.7 (7.47)	3.3 (2.09)	3.2	3.2	3.2	5.9	5.8	n.d.	5.6	5.7	5.6
**3, 4**	<1 (1.04)	<1 (1.10)	8.2 (7.54)	8.1	8.4	8.1	<1	<1	<1	<1	<1	<1
**4, 5a**	2.1 (3.63)	2.3 (4.95)	<1 (2.75)	<1	<1	1.7	2.4	2.7	1.4	1.9	1.9	1.4
**4, 5b**	1.3 (1.01)	1.8 (3.85)	<1 (1.49)	<1	<1	<1	2.3	2.7	2.0	2.1	2.1	1.9
**5a, 5b**	12.2	12.2	13.2	13.4	12.8	13.3	12.4	12.4	11.5	11.2	n.d.	n.d.
OC**H**_2_Ph						12.4					11.8	11.8

^a^ experimental error in the measured ^1^H-^1^H coupling constants was ±0.5 Hz. ^b^ weight average of the values calculated with the Haasnoot–Altona equation [42] from the corresponding dihedral angle for each conformer and the mole fraction with which it is present in the Boltzmann population at 298 K.

**Table 4:** Experimentally observed n.O.e. and H-H calculated distances for compounds **11*R***, **11*S***, and **12*R***.

	11*R*		11*S*		12*R*	
Proton Pair	n.O.e.	H-H (Å) ^a^	n.O.e.	H-H (Å) ^a^	n.O.e.	H-H (Å) ^a^
**H^acetal^-H^2^**	+	3.46	−	3.98	+	4.82
**H^acetal^-H^3^**	+	2.90	−	3.81	+	2.78
**H^acetal^-H^4^**	−	4.49	−	4.28	−	3.14
**H^acetal^-H^2^′**	−	3.68	−	3.27	−	3.78
**H^acetal^-H^6^′**	+	2.47	+	3.04	+	2.35
**H^3^-H^4^**	−	2.80	−	2.81	+	2.39

^a^ weight average of the calculated distances in the conformers as a function of their abundance in the population of the compound.

## Data Availability

The data presented in this study are available within the article and in the Supplementary Materials.

## Supplementary Materials

The following are available online. ^1^H and ^13^C-NMR spectra of compounds **11*R***, **11*S***, **12*R***, **15*R***, **16*R***, **17*R***, **18*R***, **18*S***, **19*R***, **19*S***, **20*R***, **20*S***; Energies, relative energies and Boltzmann population distribution at 298 K of conformers of the compounds **12*R*** (Table S1) and 12S (Table S2), calculated using B3LYP/6-31G* and SM8 solution model for DMSO; energies, relative energies and Boltzmann population distribution at 298 K of conformers of the compounds 11R (Table S3) and 11S (Table S4), calculated using B3LYP/6-31G* and SM8 solution model for CHCl3.

## Sample Availability

Samples of all compounds are not available from the authors.
