# Supplementary material for: Clarifying the Use of Benzylidene Protecting Group for D-(+)-Ribono-1,4-Lactone, an Essential Building Block in the Synthesis of C-Nucleosides
Source: Molecules. 2021 Oct 26;26(21):6447. doi: 10.3390/molecules26216447 (PMC8587313; doi:10.3390/molecules26216447)
Supplement: Supplementary file 1 [file molecules-26-06447-s001.zip › molecules-1410181-supplementary.pdf]

Supplemental Material

Clarifying the use of benzylidene protecting group for D-(+)-ribono-1,4-lactone, an essential building block in the synthesis of C-nucleosides.

Silvana Casati[1], Paola Rota[2], Pietro Allevi[2], Alessandra Mingione[1], Roberta Ottria[1*], Pierangela Ciuffreda[1]

[1] *Dipartimento di Scienze Biomediche e Cliniche "Luigi Sacco", Università degli Studi di Milano, Via G.B. Grassi 74, 20157 Milano, Italy; silvana.casati@unimi.it, alessandra.mingione@unimi.it, roberta.ottria@unimi.it, pierangela.ciuffreda@unimi.it*

[2] *Dipartimento di Scienze Biomediche, Chirurgiche e Odontoiatriche, Università degli Studi di Milano, Via Saldini 50, 20122 Milano, Italy; pietro.allevi@unimi.it, paola.rota@unimi.it.*

Table of Contents

^{1}H and ^{13}C-NMR spectra Pag. 2-17

Table S1-S4 Pag. 18

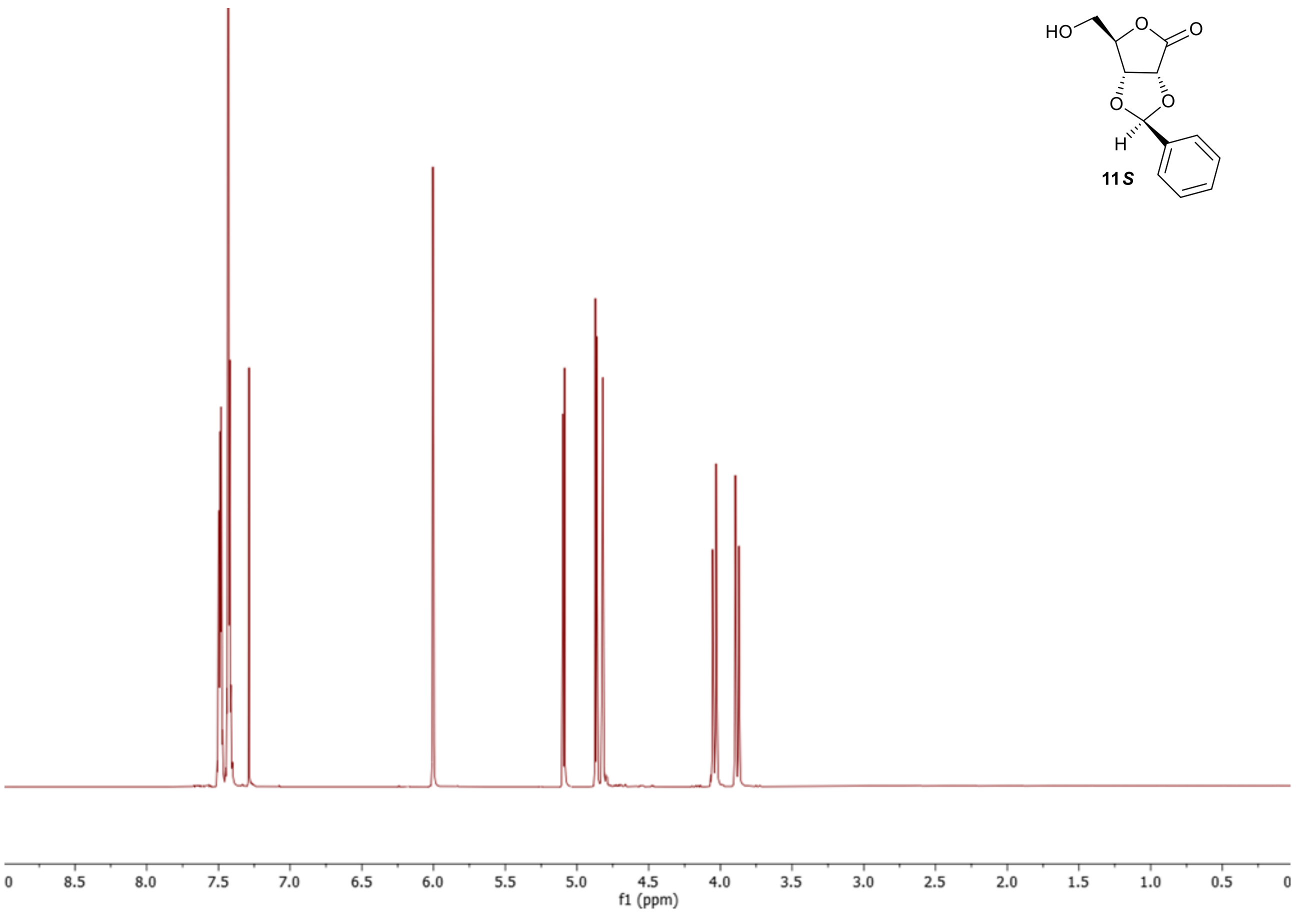
HO
O
O
O
O
H
11S
f1 (ppm)

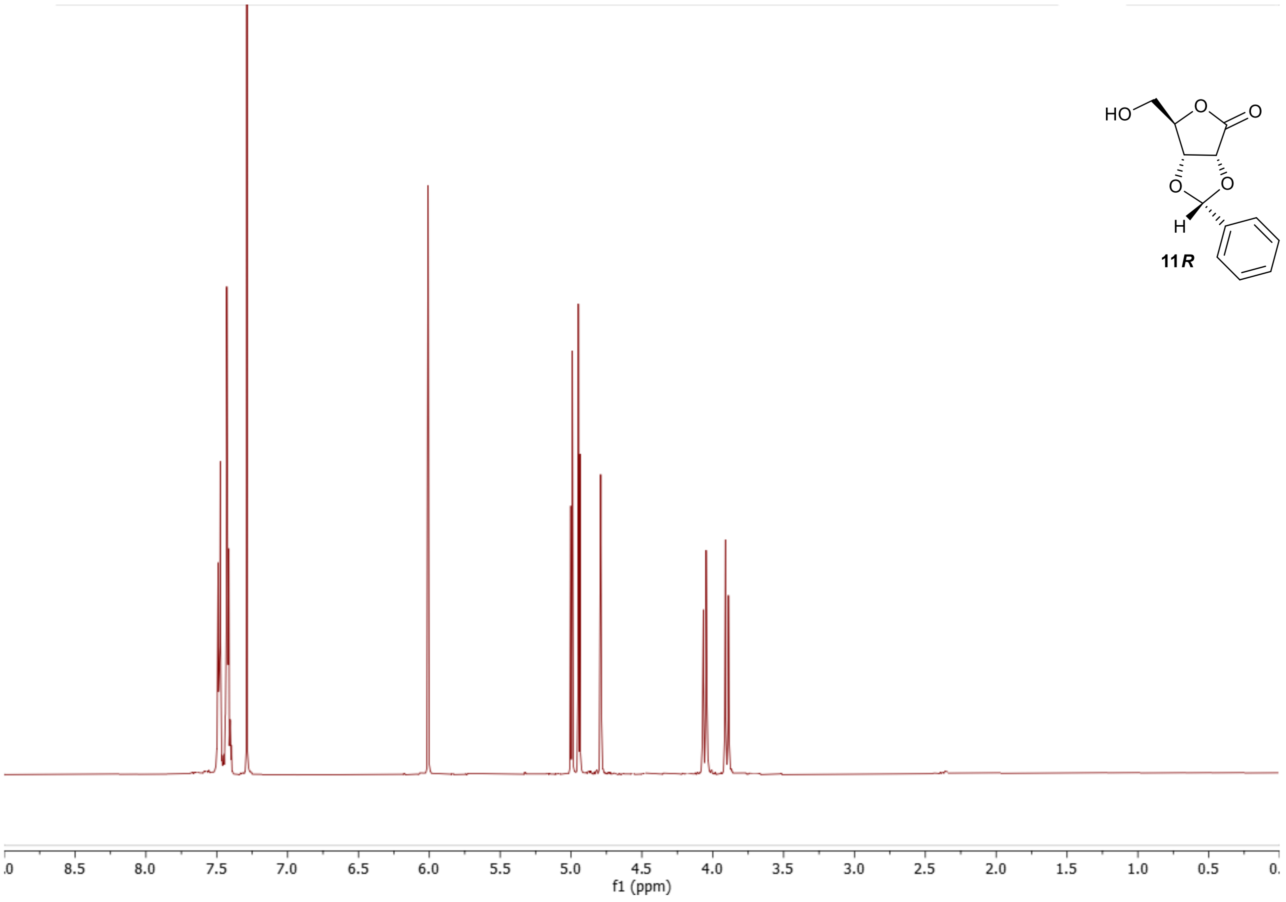
HO
O
O
O
O
H
11R
f1 (ppm)

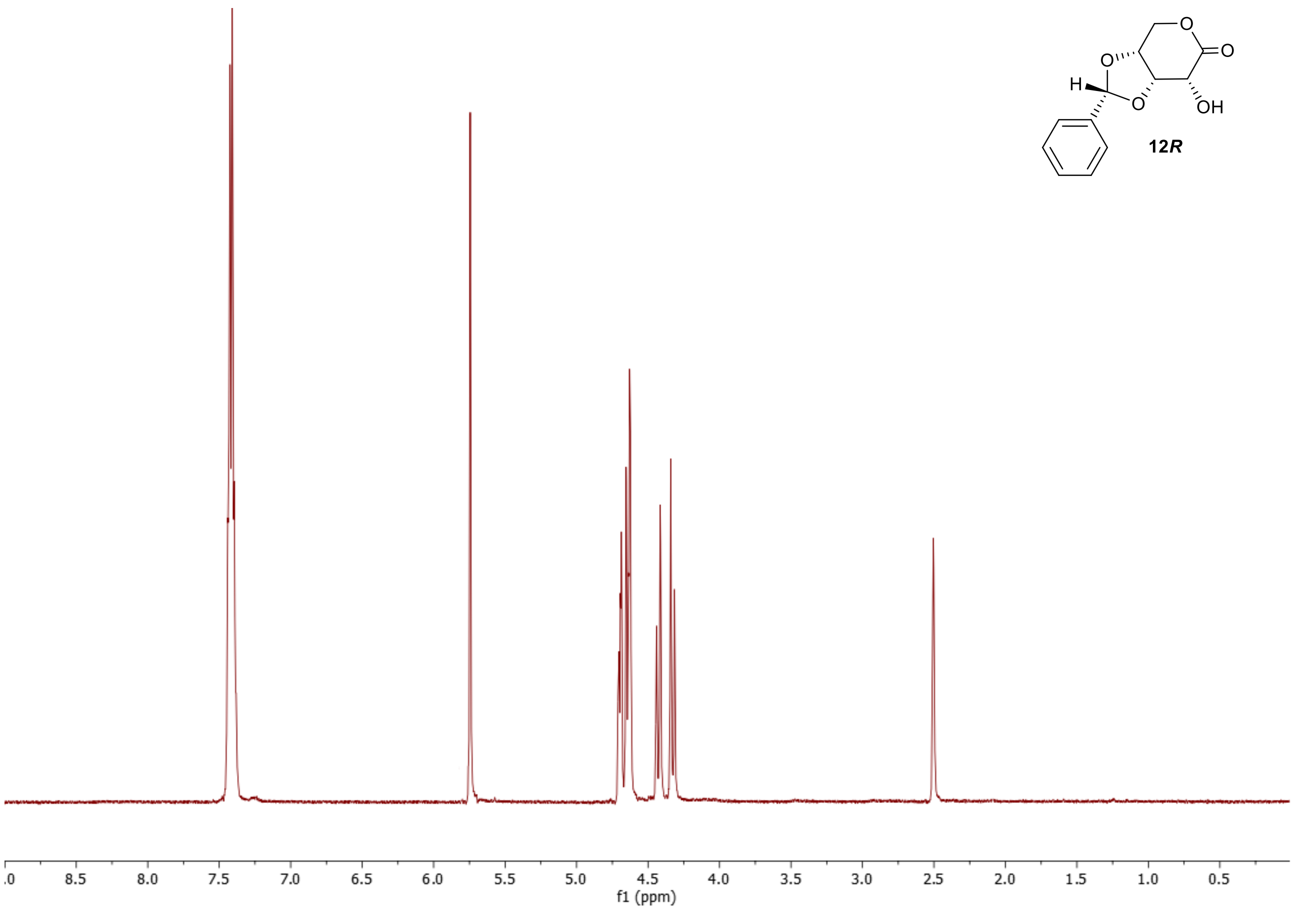

12*R*

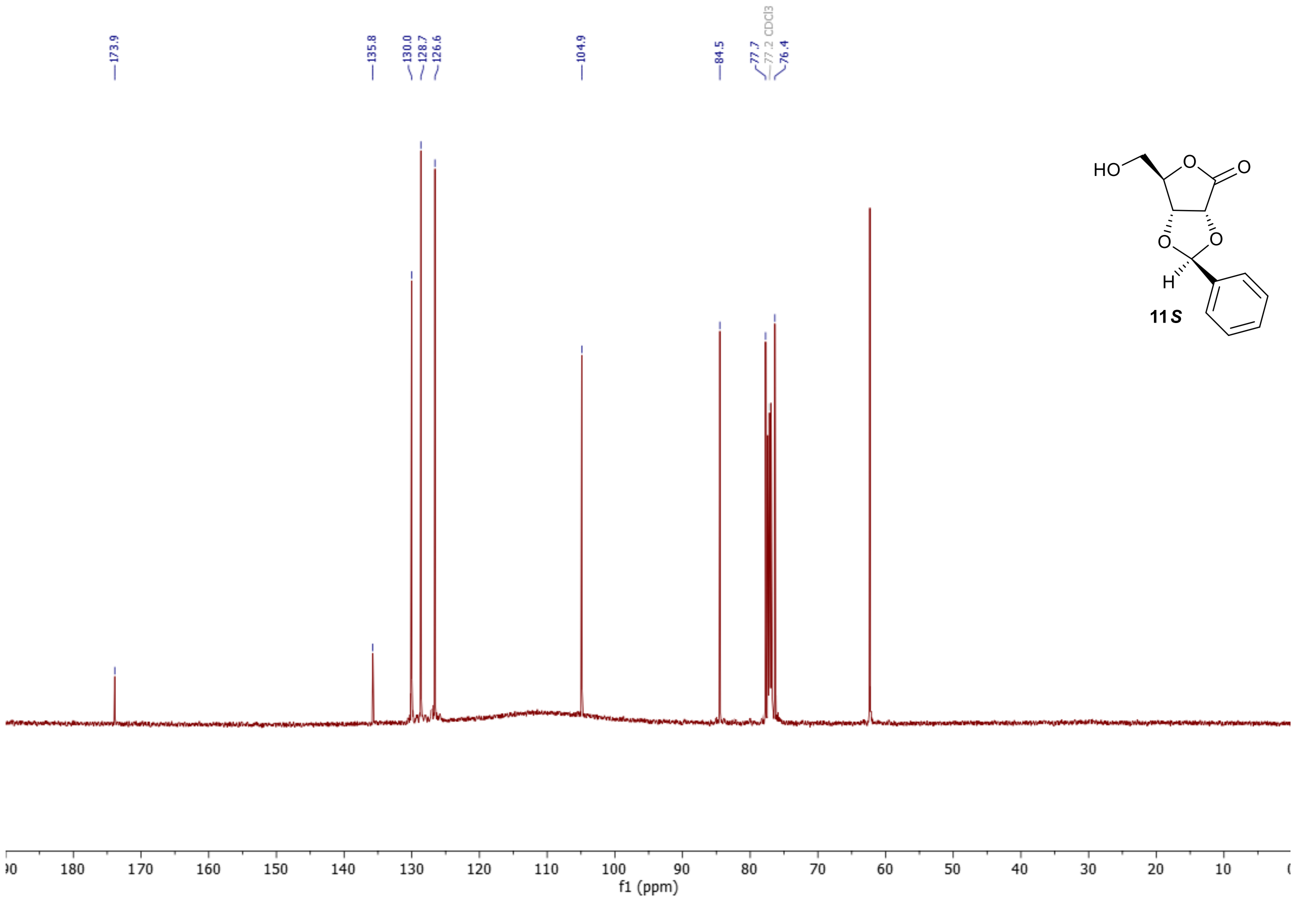
173.9
135.8
130.0
128.7
126.6
104.9
84.5
77.7
77.2 CDCl3
76.4
HO
O
O
O
O
H
f1 (ppm)

11*S*

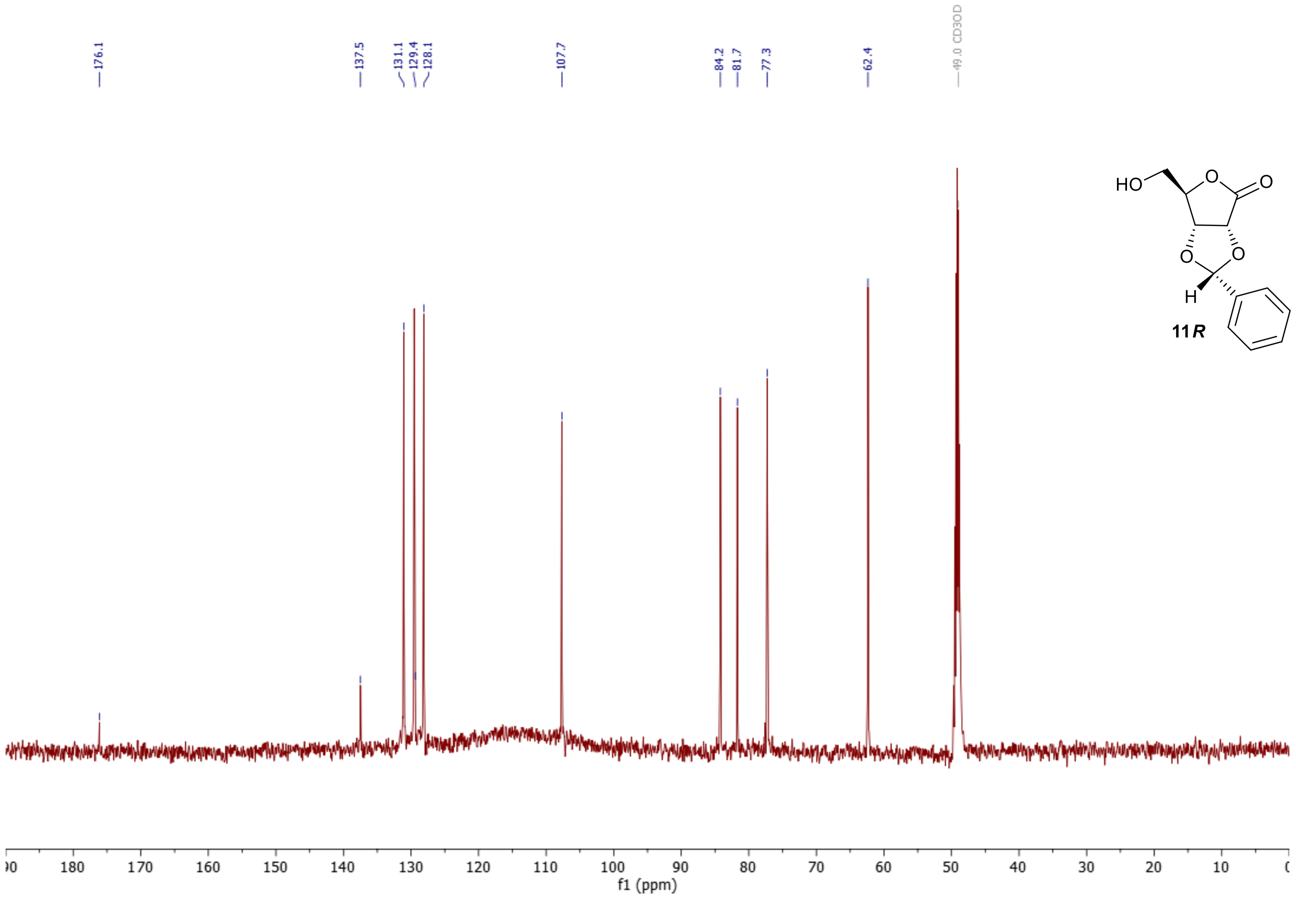
176.1
137.5
131.1
129.4
128.1
107.7
84.2
81.7
77.3
62.4
49.0 CD3OD
HO
O
O
O
O
H
f1 (ppm)

11*R*

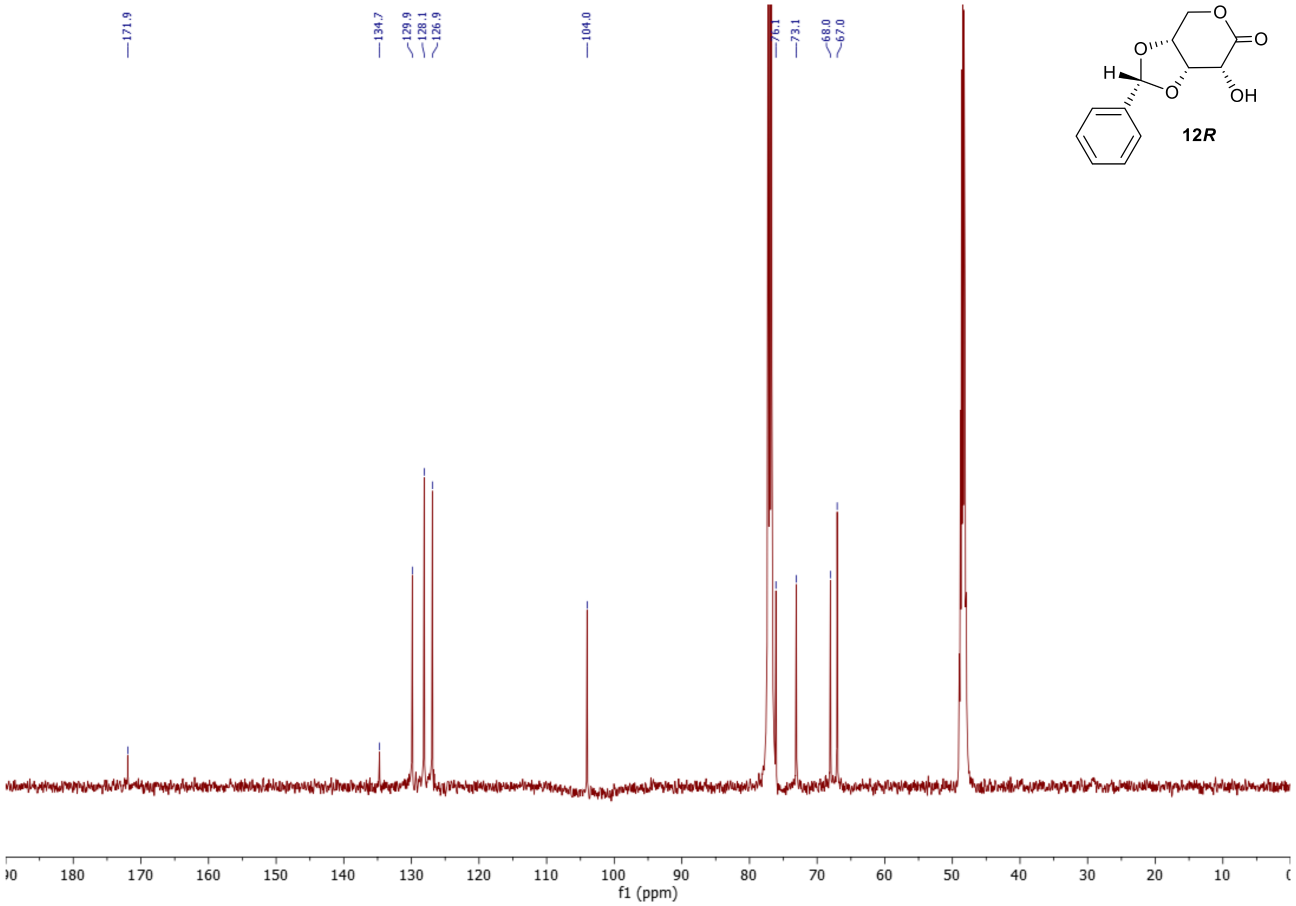
171.9
134.7
129.9
128.1
126.9
104.0
76.1
73.1
68.0
67.0
180
170
160
150
140
130
120
110
100
90
80
70
60
50
40
30
20
10
f1 (ppm)
O
O
O
OH
H
12*R*

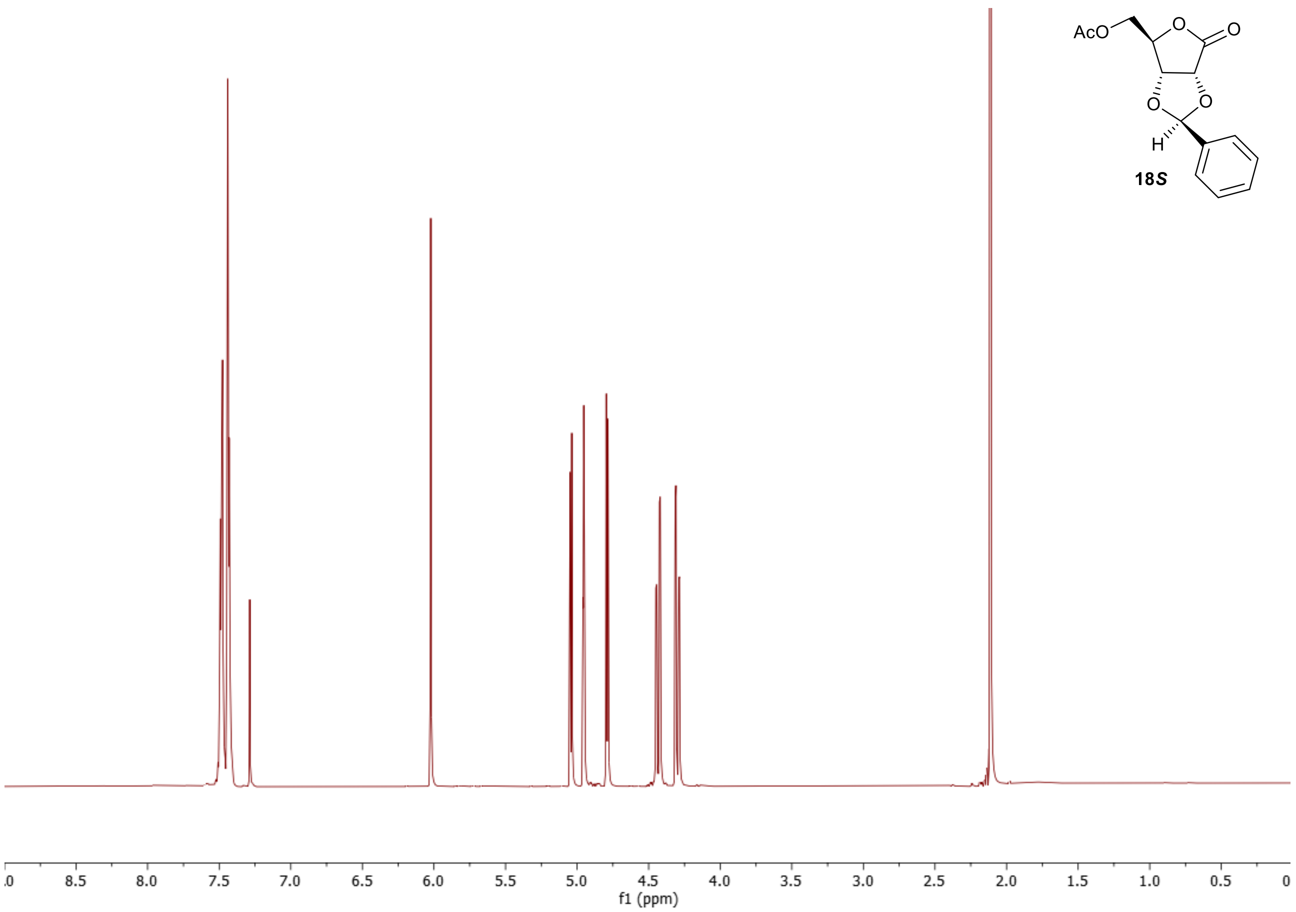
AcO
O
O
O
O
H
18S
9.0
8.5
8.0
7.5
7.0
6.5
6.0
5.5
5.0
4.5
4.0
3.5
3.0
2.5
2.0
1.5
1.0
0.5
0
f1 (ppm)

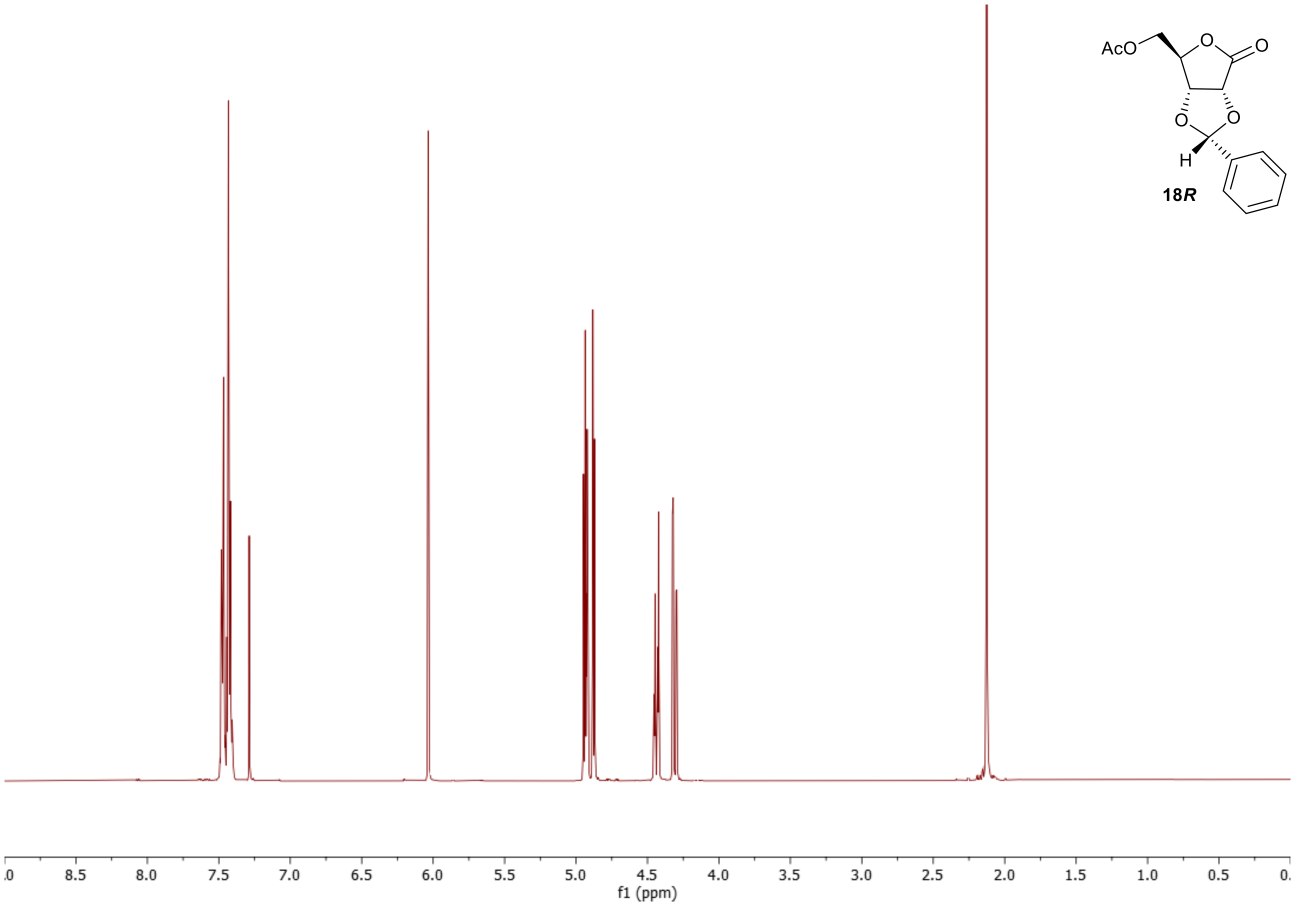
AcO
O
O
O
O
H
18R
8.5
8.0
7.5
7.0
6.5
6.0
5.5
5.0
4.5
4.0
3.5
3.0
2.5
2.0
1.5
1.0
0.5
f1 (ppm)

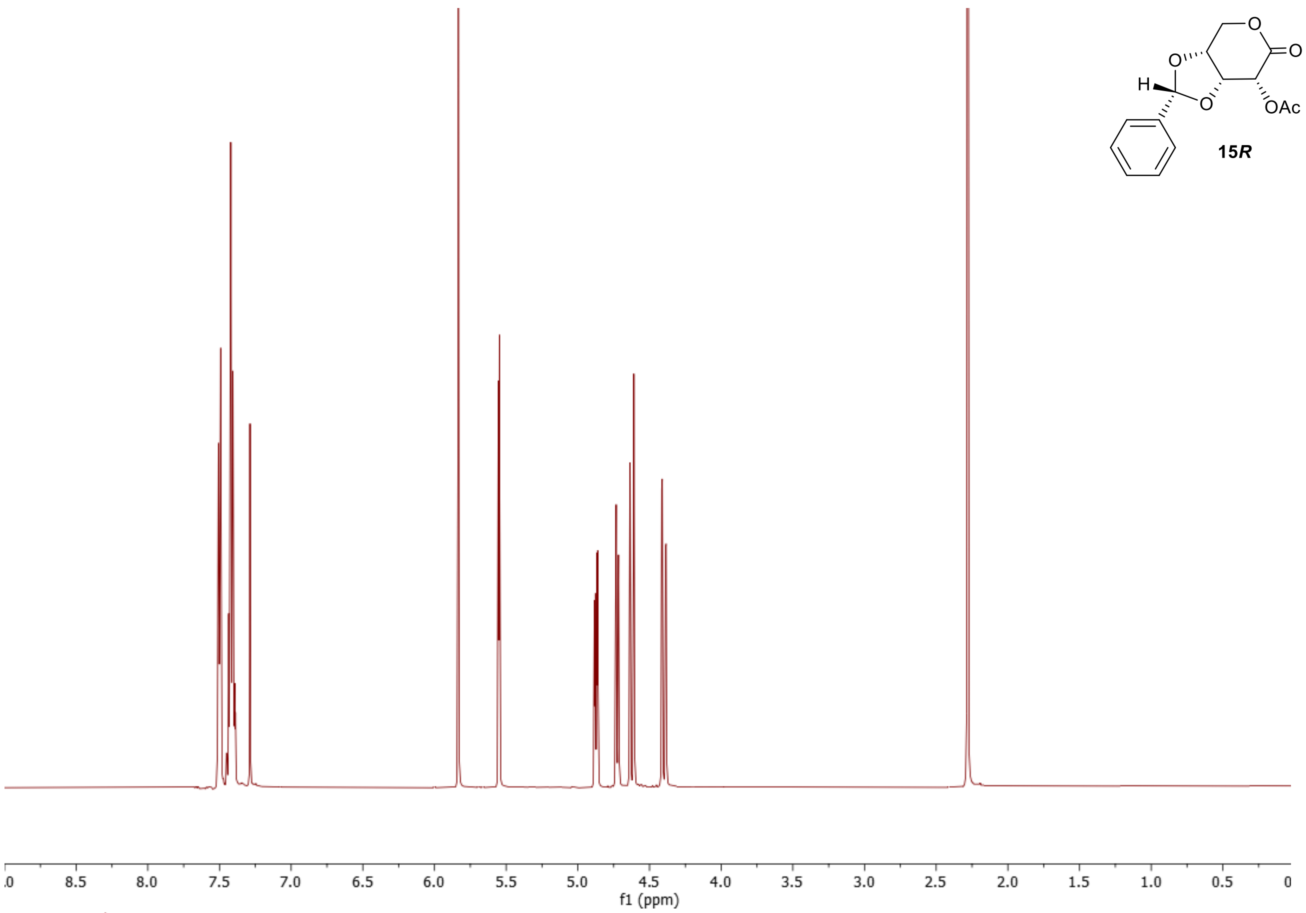
15*R*
OAc
f1 (ppm)

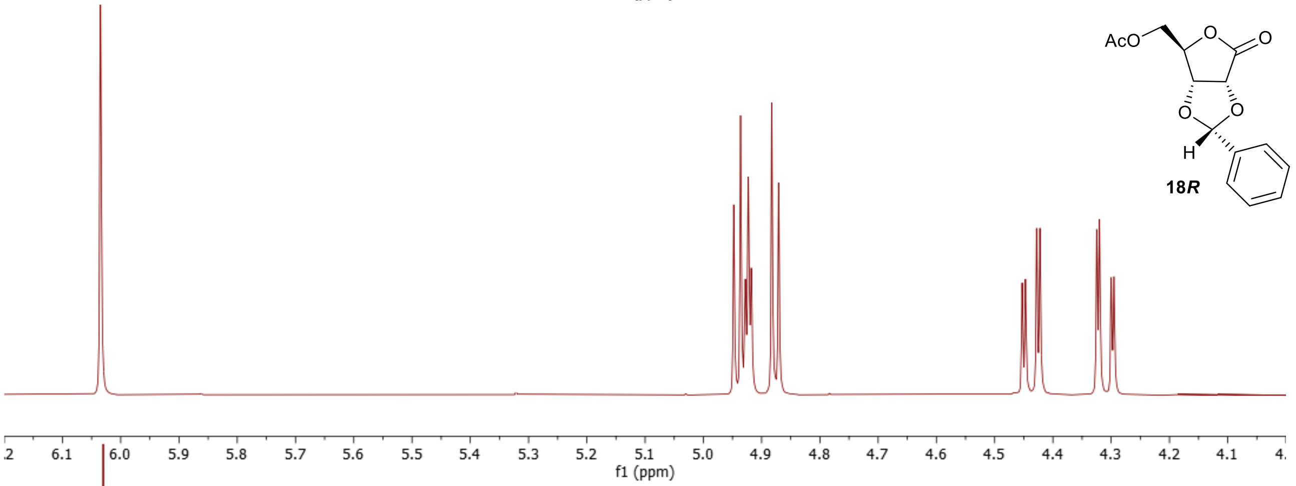
AcO
18*R*
f1 (ppm)

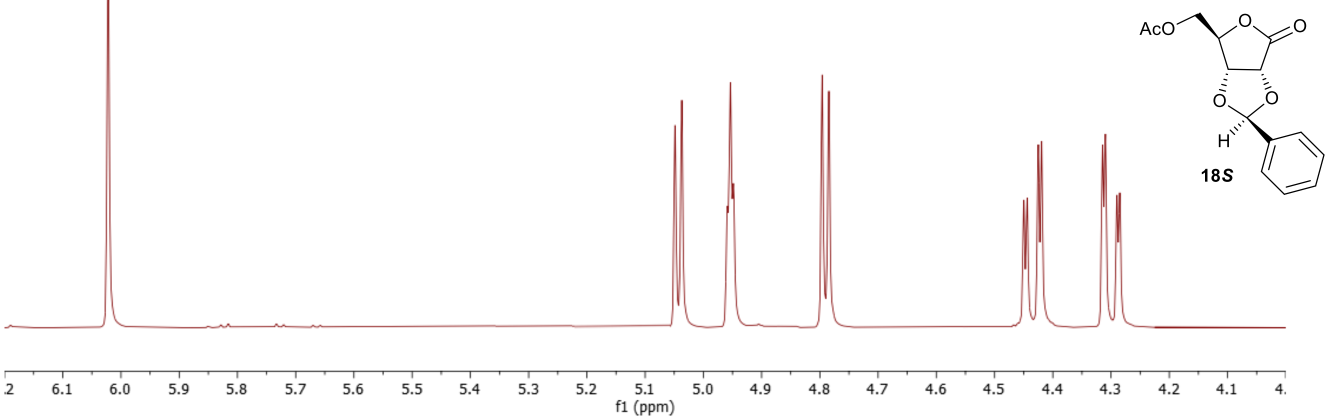
AcO
18*S*
f1 (ppm)

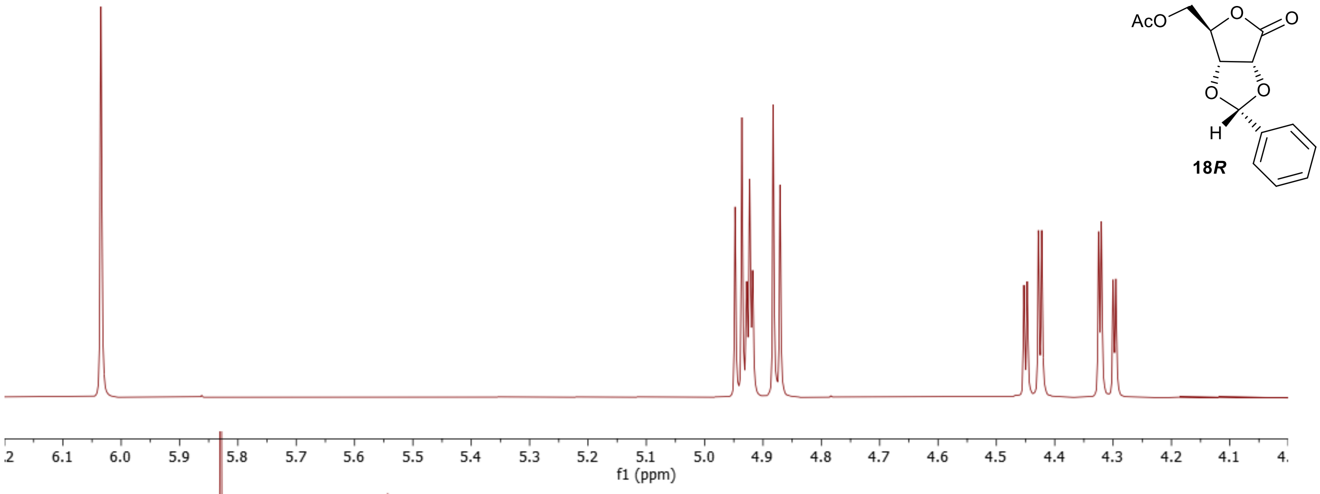
AcO
O
O
O
O
H
18*R*
f1 (ppm)

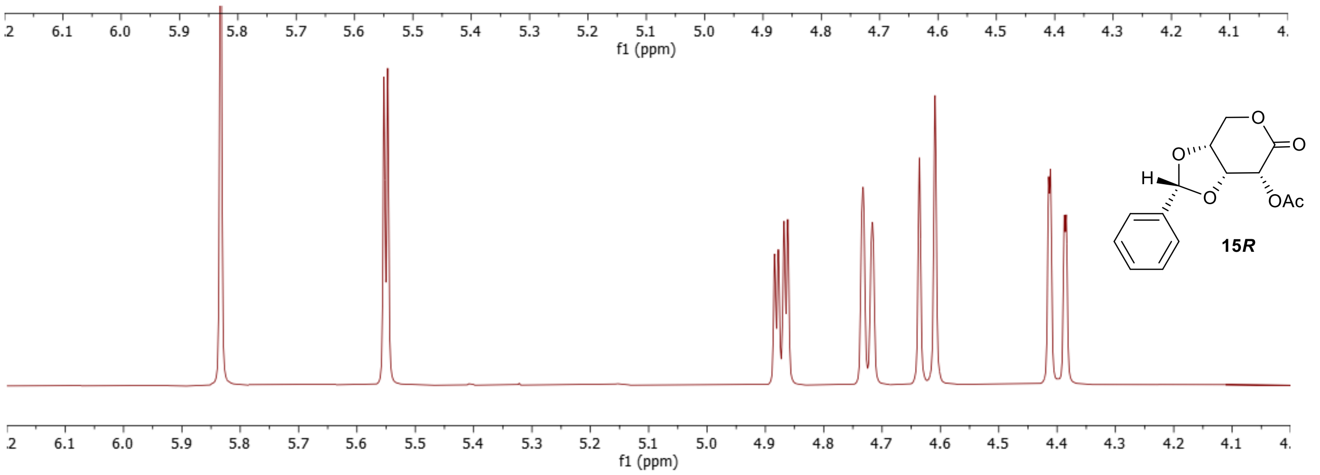
O
O
O
O
H
OAc
15*R*
f1 (ppm)

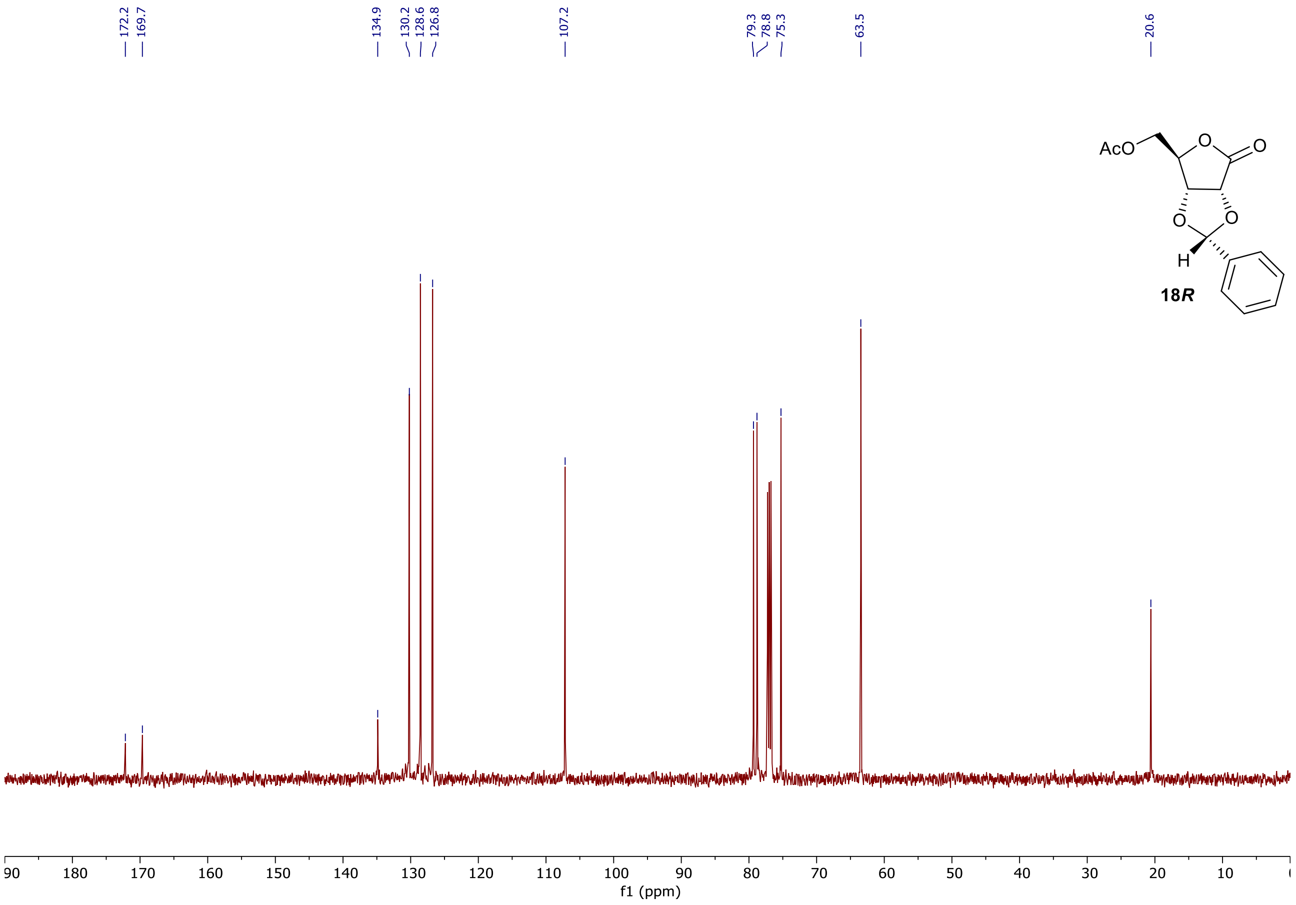
172.2
169.7
134.9
130.2
128.6
126.8
107.2
79.3
78.8
75.3
63.5
20.6
AcO
O
O
O
O
H
18R
f1 (ppm)

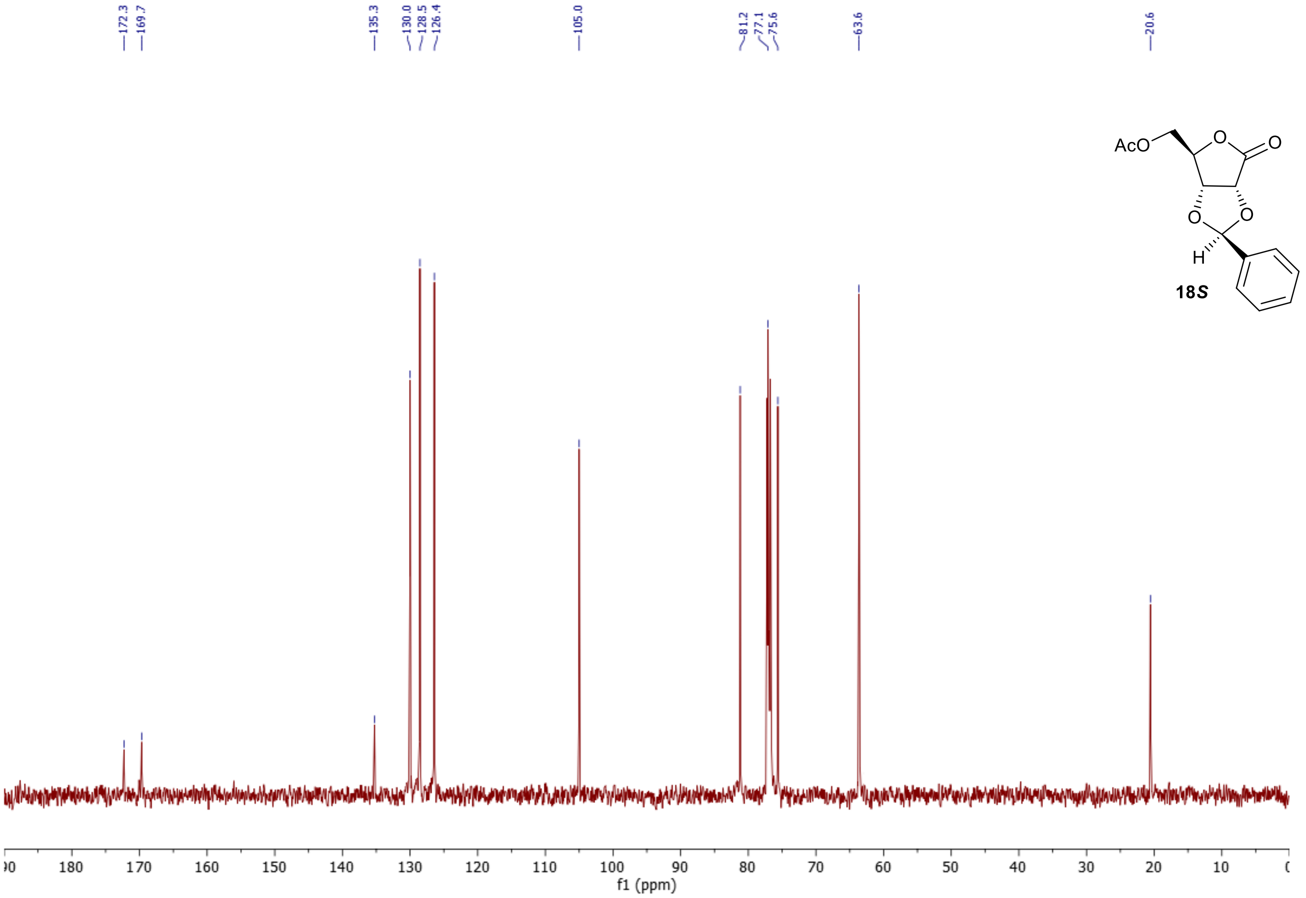
172.3
169.7
135.3
130.0
128.5
126.4
105.0
81.2
77.1
75.6
63.6
20.6
AcO
O
O
O
O
H
18S
f1 (ppm)

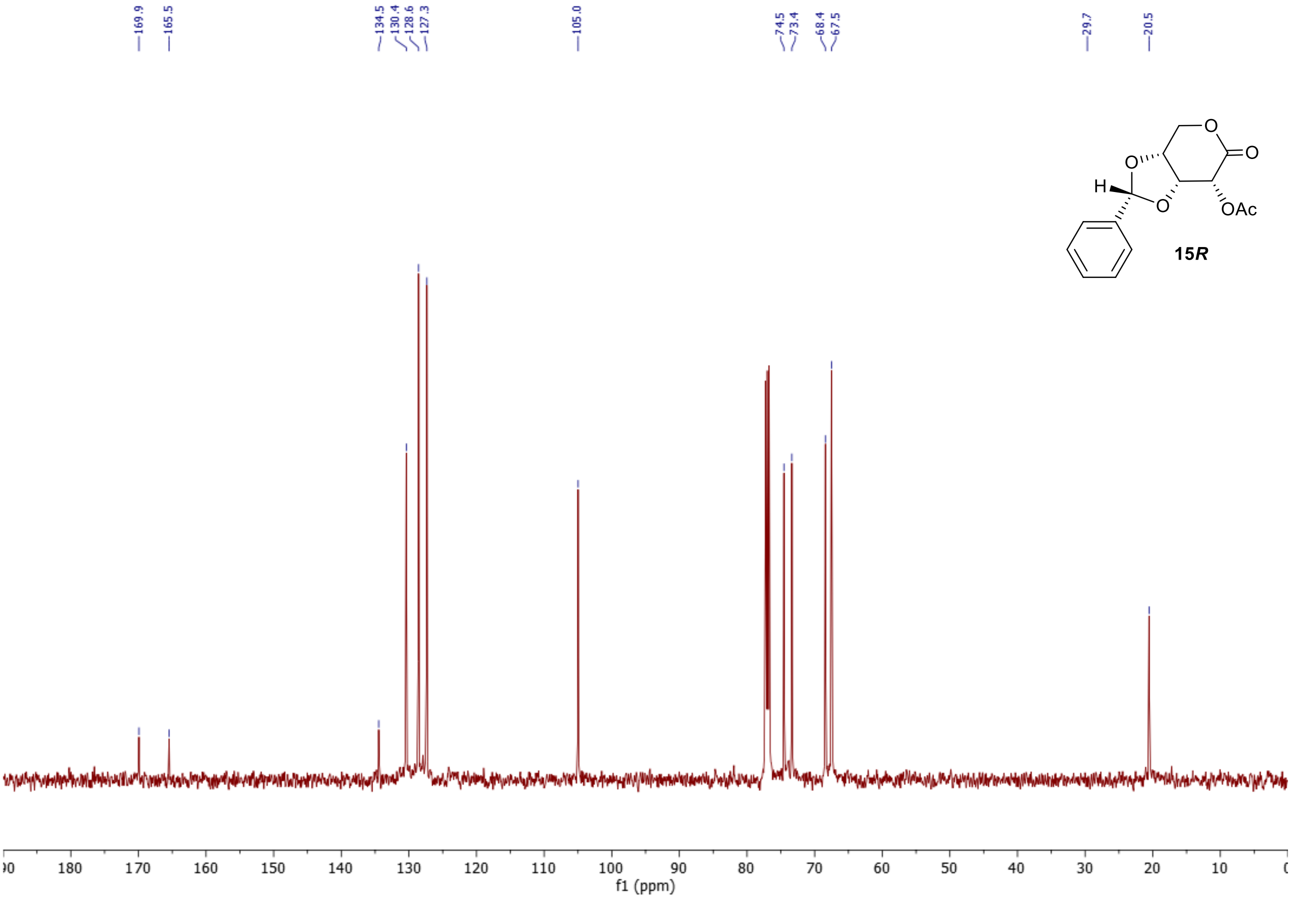
169.9
165.5
134.5
130.4
128.6
127.3
105.0
74.5
73.4
68.4
67.5
29.7
20.5
O
O
H
O
O
OAc
15R
180
170
160
150
140
130
120
110
100
90
80
70
60
50
40
30
20
10
f1 (ppm)

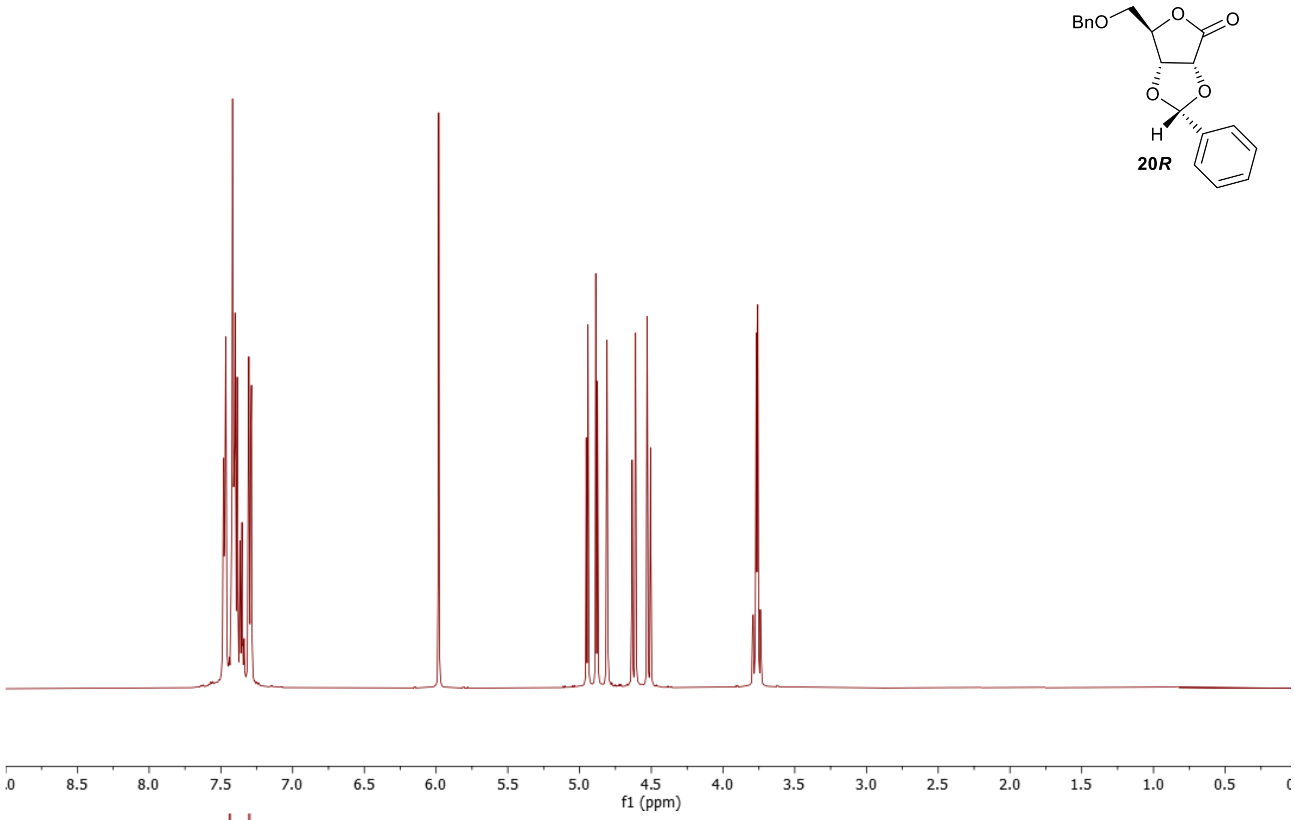
BnO
O
O
O
O
H
8.5
8.0
7.5
7.0
6.5
6.0
5.5
5.0
4.5
4.0
3.5
3.0
2.5
2.0
1.5
1.0
0.5
f1 (ppm)

20*R*

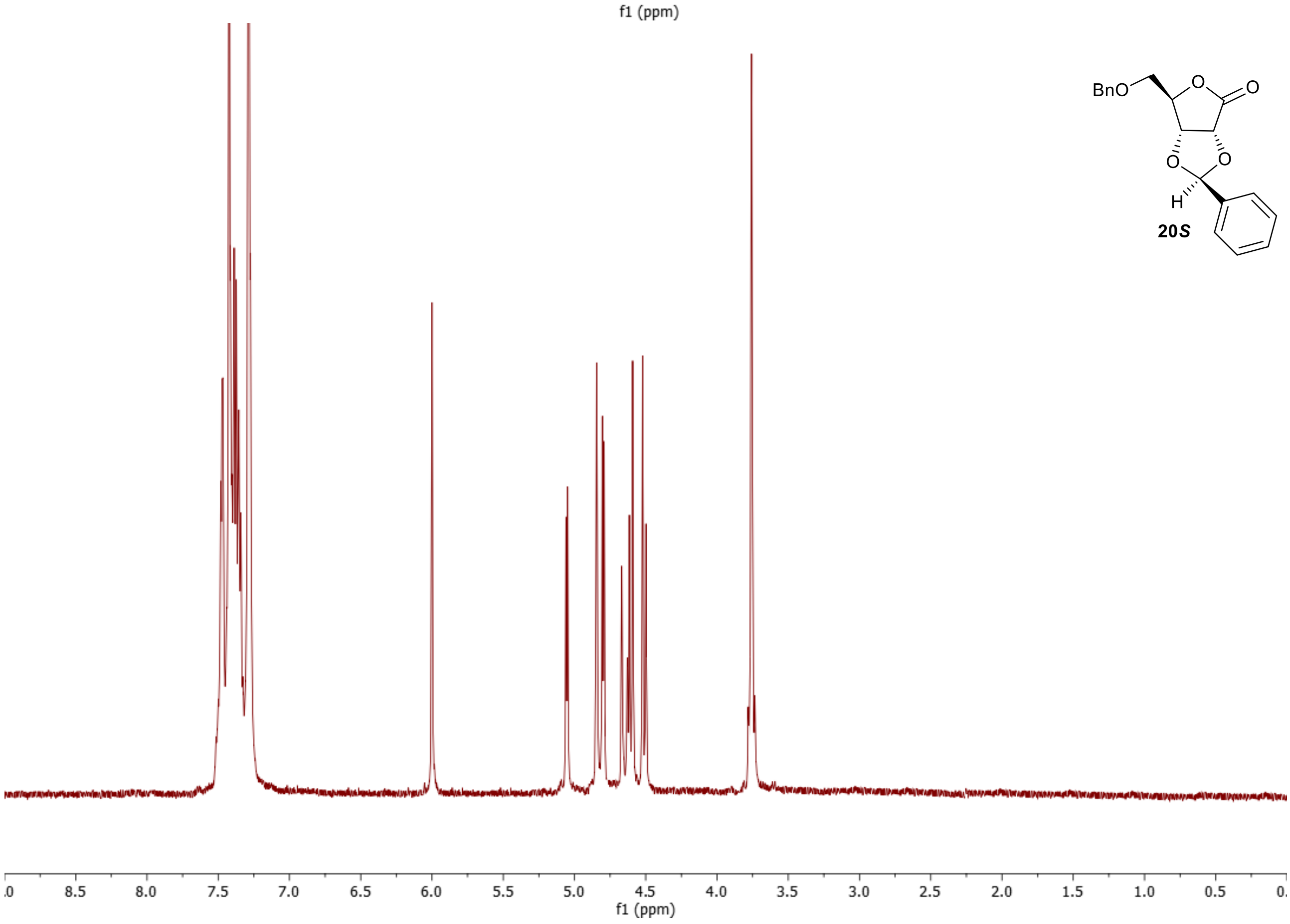
BnO
O
O
O
O
H
8.5
8.0
7.5
7.0
6.5
6.0
5.5
5.0
4.5
4.0
3.5
3.0
2.5
2.0
1.5
1.0
0.5
f1 (ppm)

20*S*

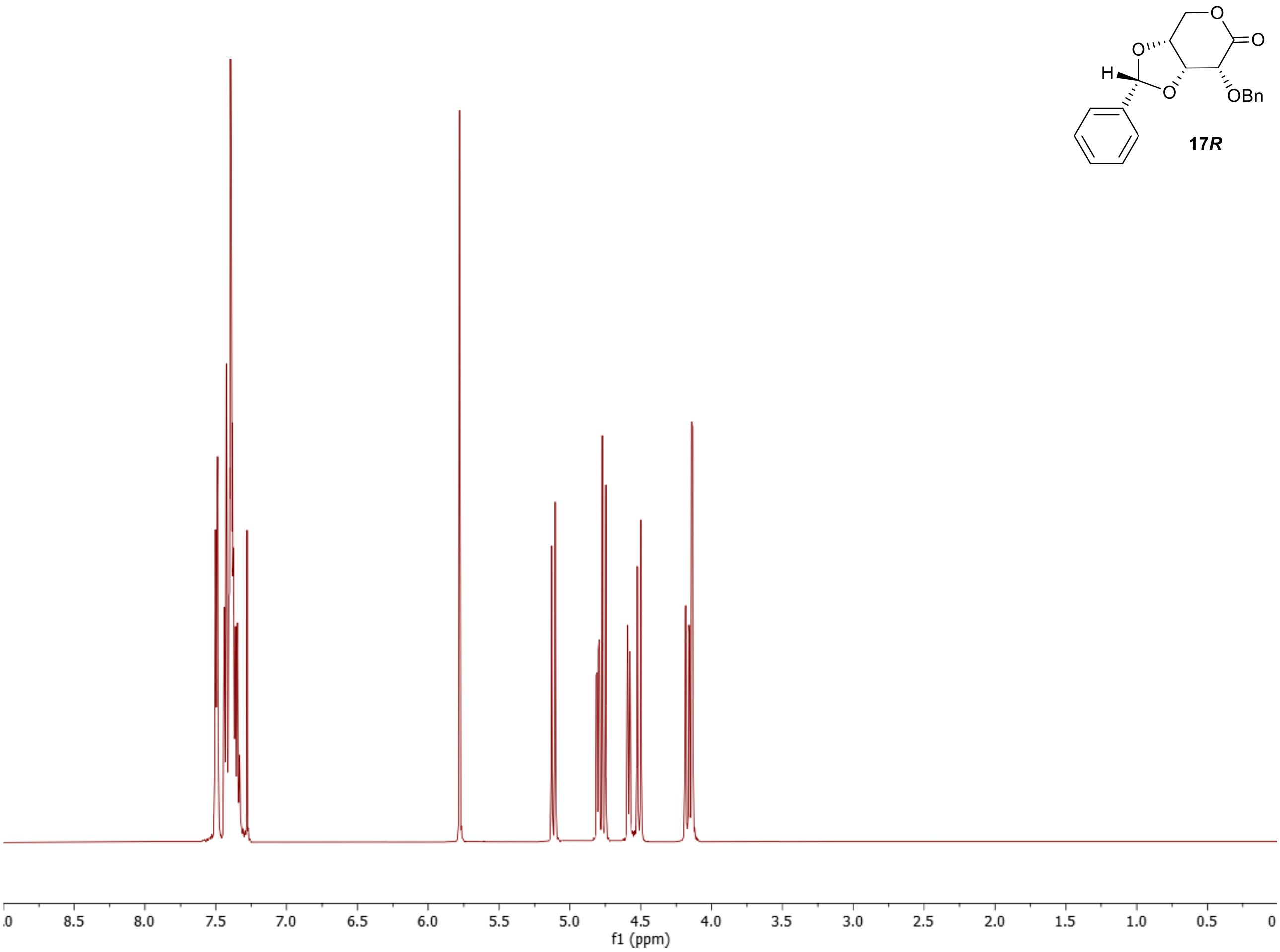
O
O
H
O
O
OBn
17R
.0
8.5
8.0
7.5
7.0
6.5
6.0
5.5
5.0
4.5
4.0
3.5
3.0
2.5
2.0
1.5
1.0
0.5
0
f1 (ppm)

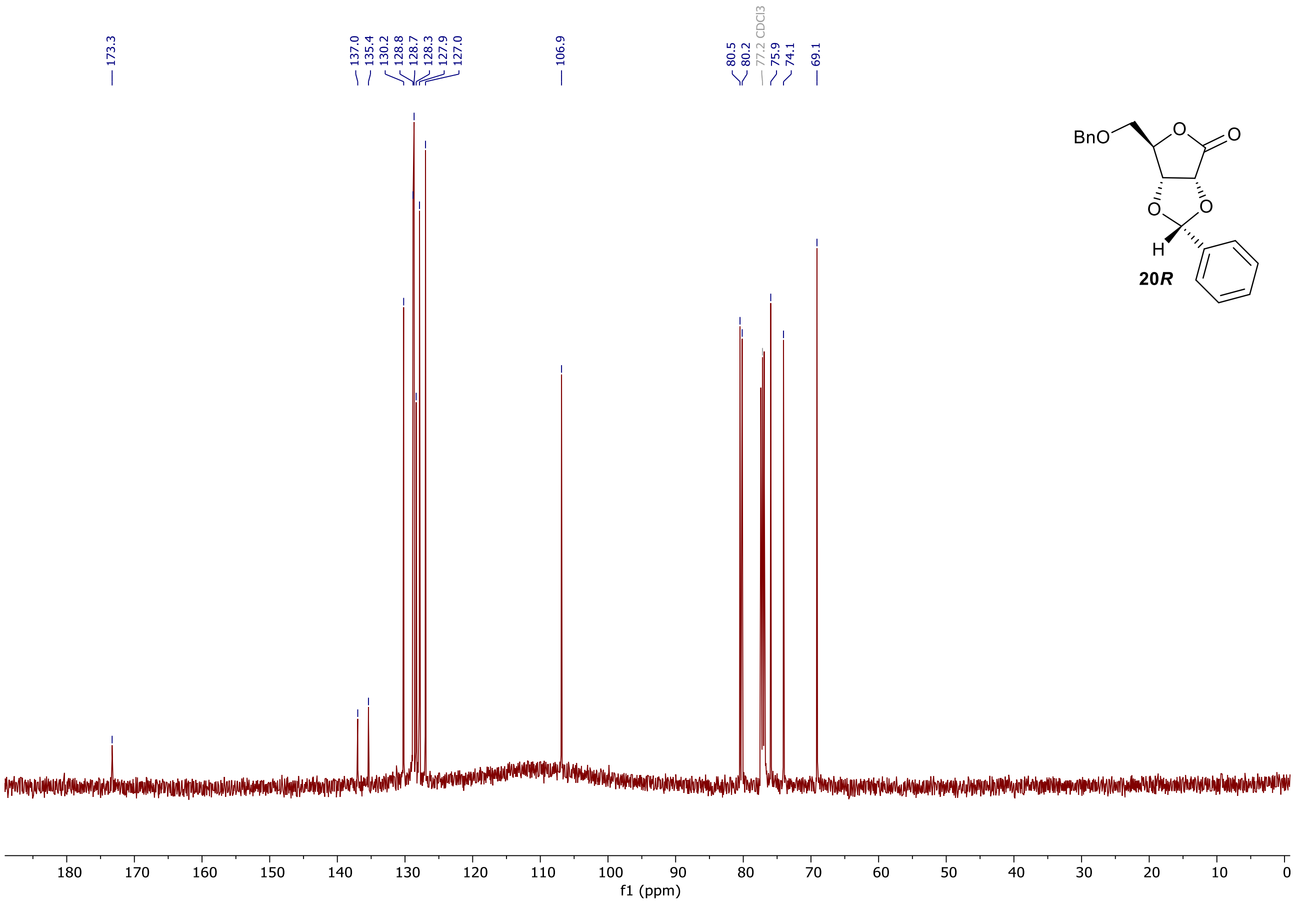

173.3
137.0
135.4
130.2
128.8
128.7
128.3
127.9
127.0
106.9
80.5
80.2
77.2 CDCl3
75.9
74.1
69.1
BnO
20R
180 170 160 150 140 130 120 110 100 90 80 70 60 50 40 30 20 10 0
f1 (ppm)

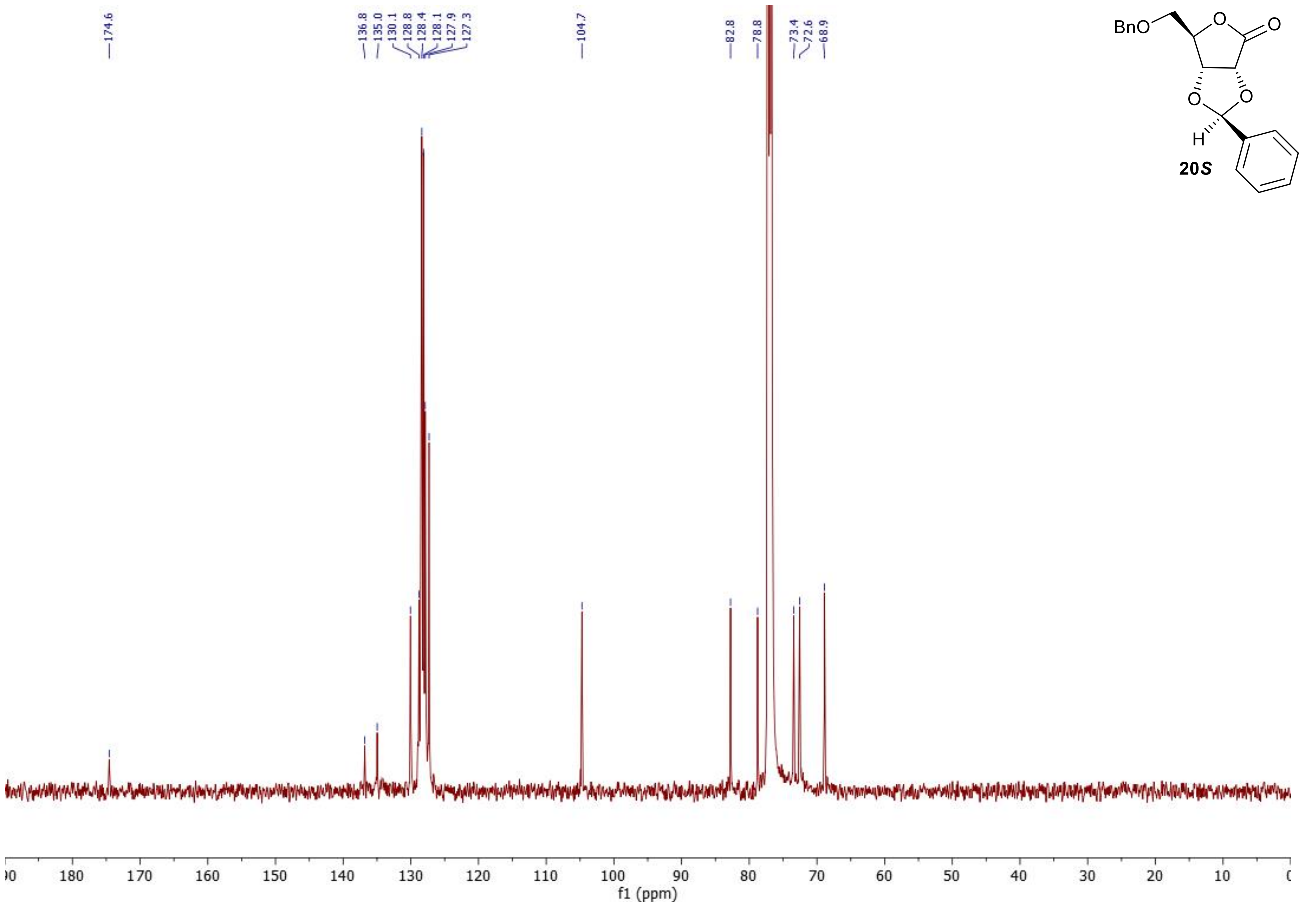

174.6
136.8
135.0
130.1
128.8
128.4
128.1
127.9
127.3
104.7
82.8
78.8
73.4
72.6
68.9
BnO
20S
180 170 160 150 140 130 120 110 100 90 80 70 60 50 40 30 20 10
f1 (ppm)

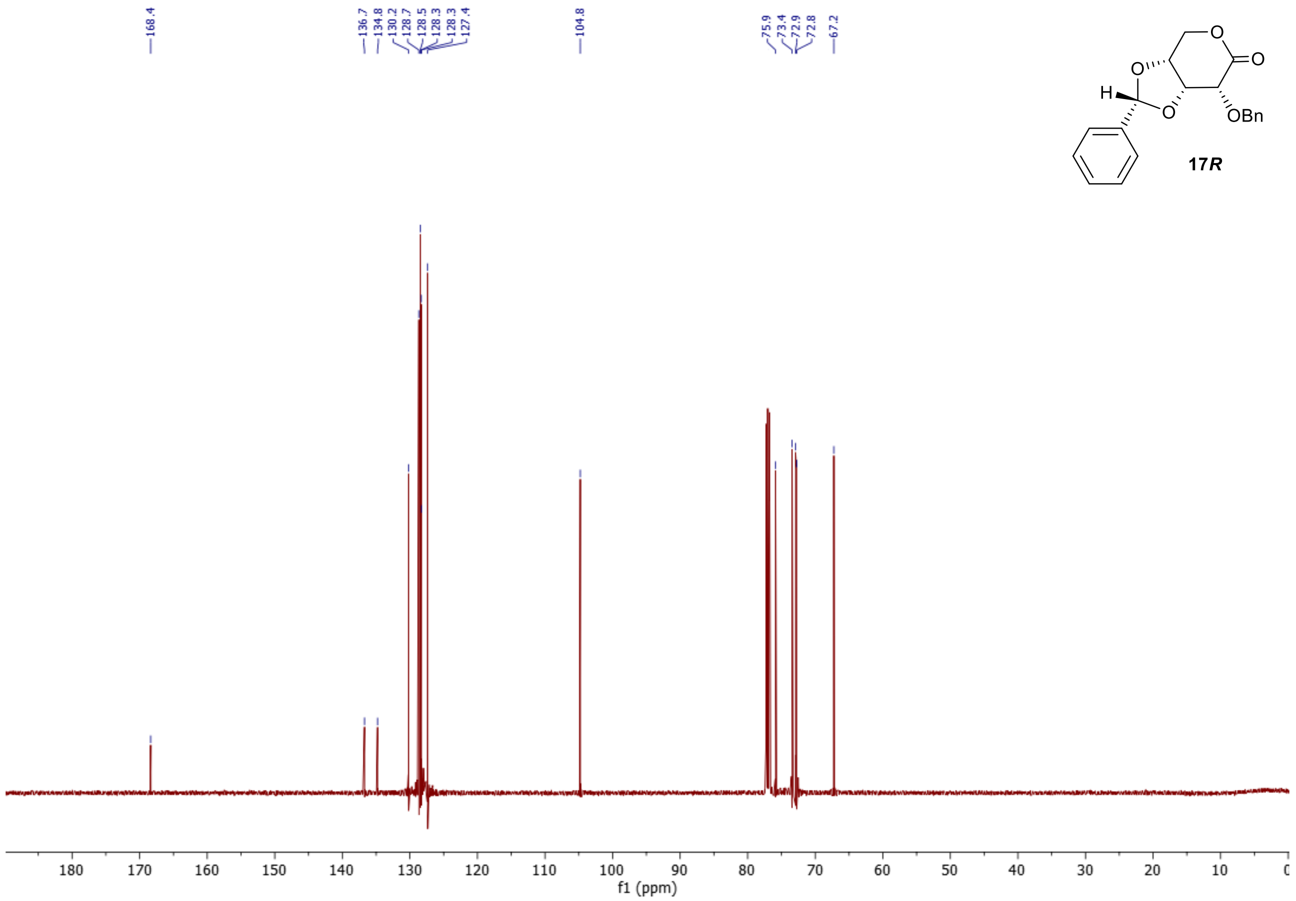
168.4
136.7
134.8
130.2
128.7
128.5
128.3
128.3
127.4
104.8
75.9
73.4
72.9
72.8
67.2
O
O
O
O
H
OBn
17*R*
180 170 160 150 140 130 120 110 100 90 80 70 60 50 40 30 20 10 0
f1 (ppm)

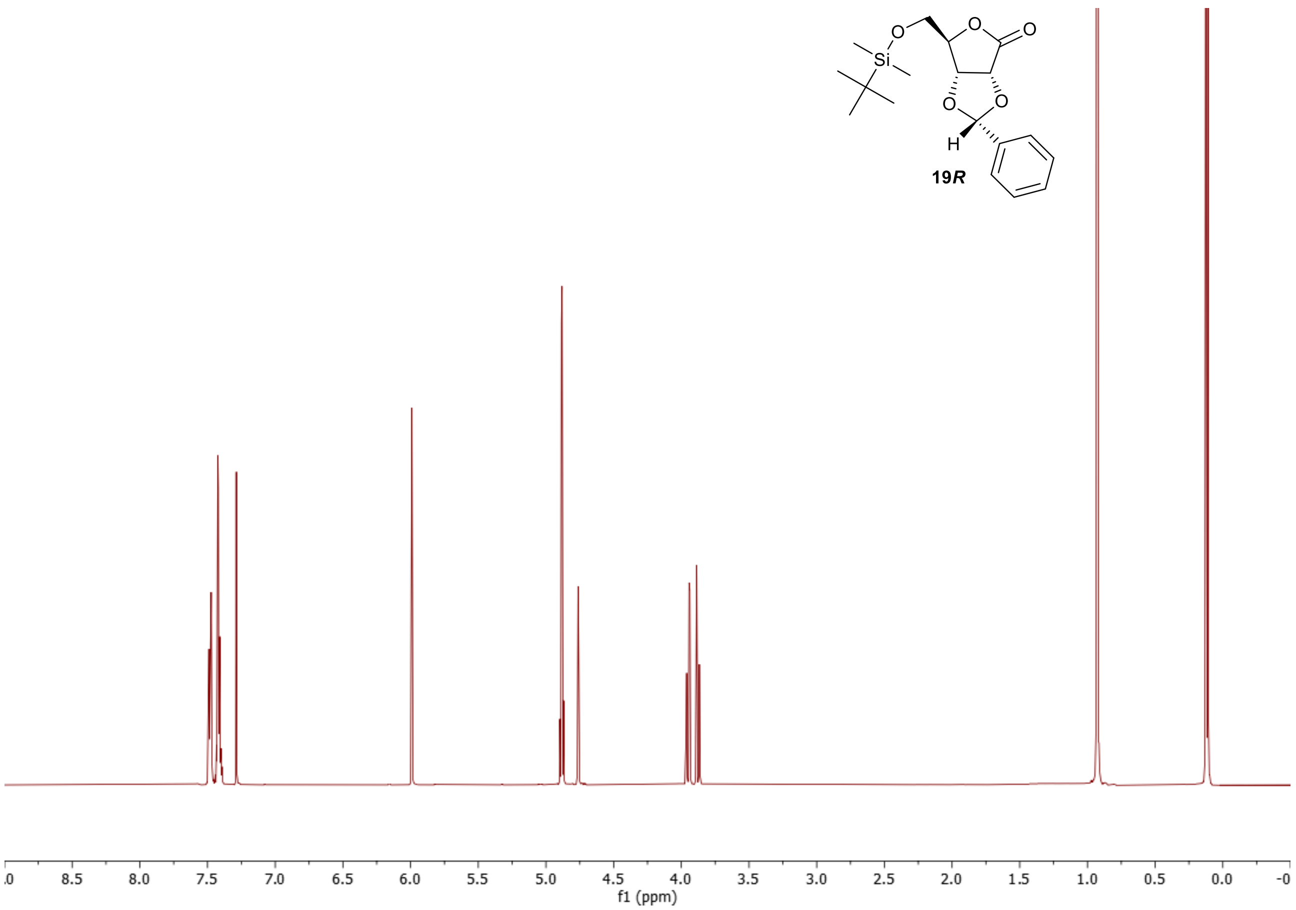

19R
f1 (ppm)

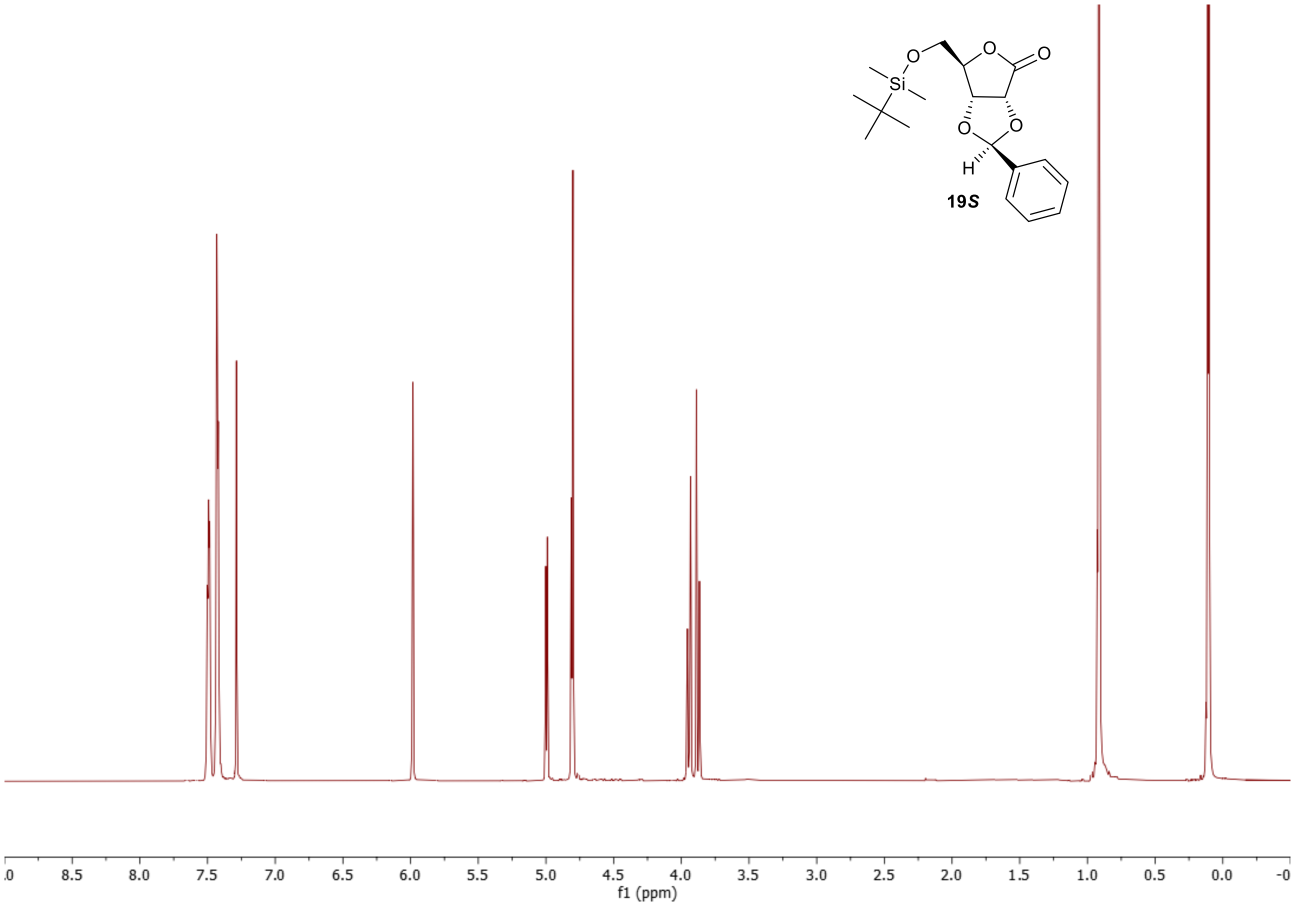

19S
f1 (ppm)

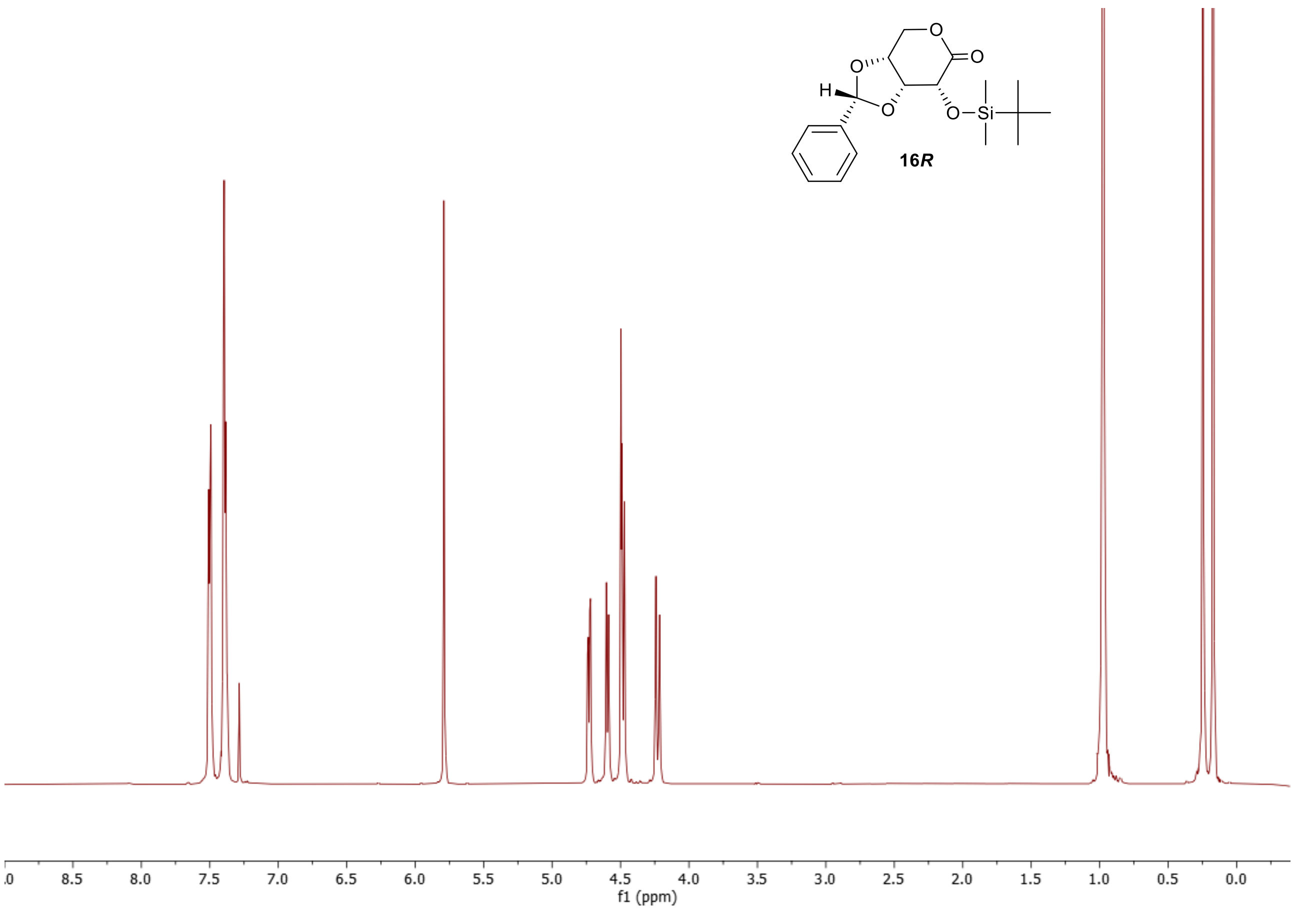
16*R*
f1 (ppm)
.0 8.5 8.0 7.5 7.0 6.5 6.0 5.5 5.0 4.5 4.0 3.5 3.0 2.5 2.0 1.5 1.0 0.5 0.0

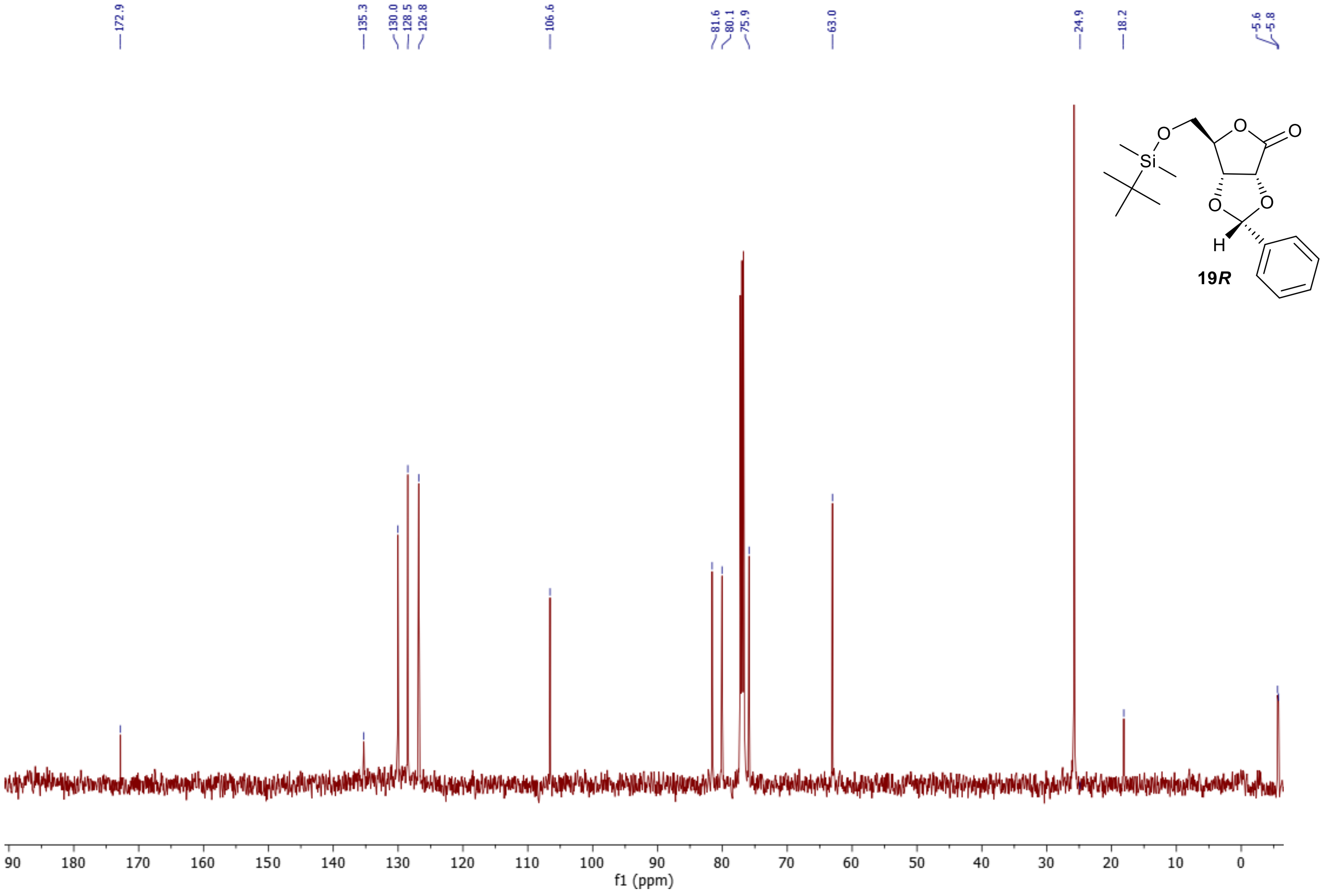
172.9
135.3
130.0
128.5
126.8
106.6
81.6
80.1
75.9
63.0
24.9
18.2
-5.6
-5.8
19*R*
f1 (ppm)
90 180 170 160 150 140 130 120 110 100 90 80 70 60 50 40 30 20 10 0

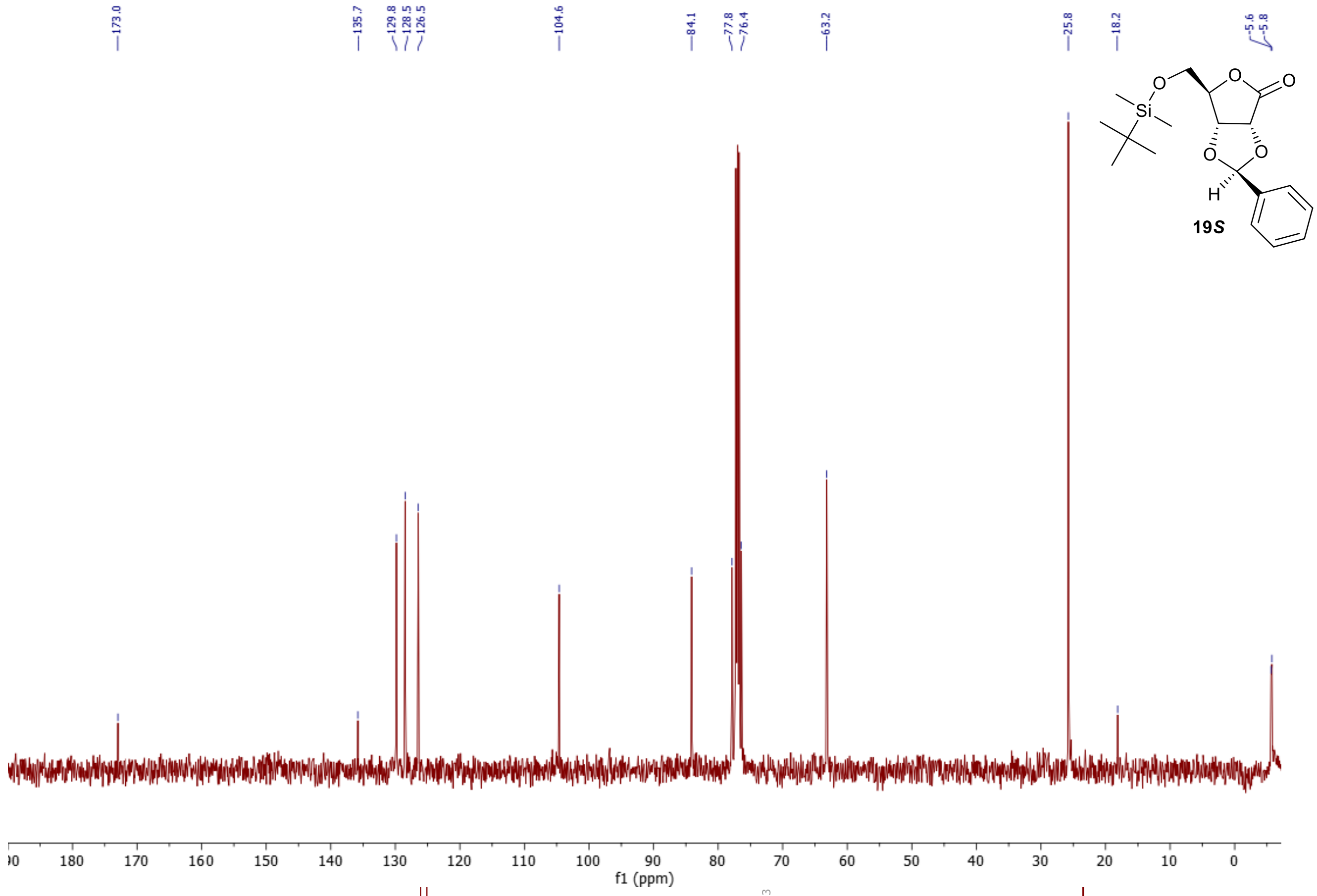
173.0
135.7
129.8
128.5
126.5
104.6
84.1
77.8
76.4
63.2
25.8
18.2
-5.6
-5.8
19*S*
f1 (ppm)

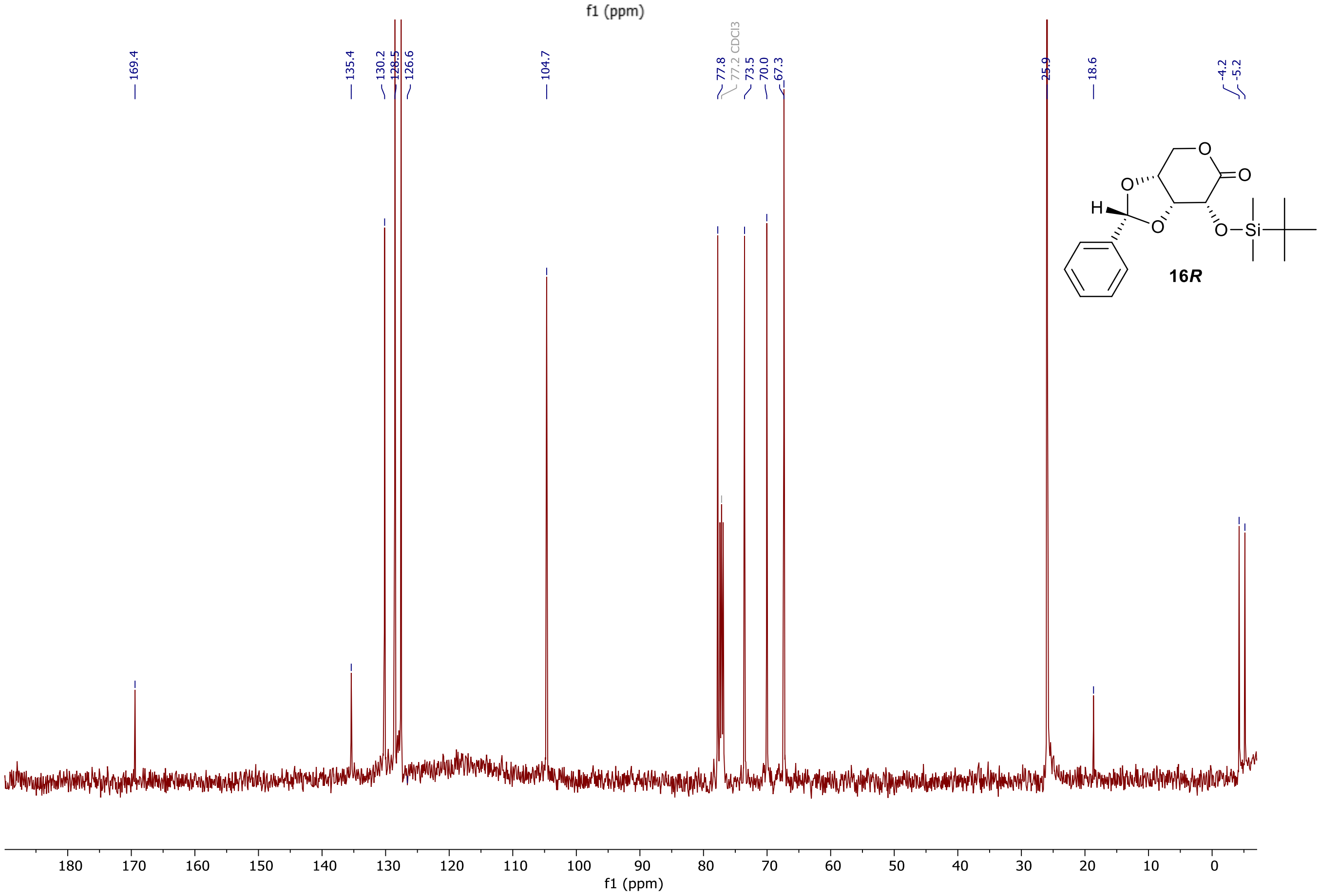
169.4
135.4
130.2
128.5
126.6
104.7
77.8
77.2 CDCl3
73.5
70.0
67.3
25.9
18.6
-4.2
-5.2
16*R*
f1 (ppm)

Table S1. Energies (E), relative energies (ΔE) in kJ/mol and Boltzmann population distribution at 298 K of conformers of the compound **12*R***, in a range of 50 kJ/mol (12 kcal/mol), calculated with a hybrid DFT (B3LYP) approach with 6-31G* basis set using SM8 (DMSO) solvation model.

E (kJ/mol)	ΔE (kJ/mol)	% in the population
-2207062.42	0.00	99.99
-2207039.64	22.78	1.00×10^{-4}
-2207031.86	30.56	4.30×10^{-6}
-2207026.05	36.37	4.11×10^{-7}
-2207025.94	36.48	3.93×10^{-7}
-2207019.50	42.92	2.90×10^{-8}

Table S2. Energies (E), relative energies (ΔE) in kJ/mol and Boltzmann population distribution at 298 K of conformers of the compound **12*S***, in a range of 50 kJ/mol (12 kcal/mol), calculated with a hybrid DFT (B3LYP) approach with 6-31G* basis set using SM8 (DMSO) solvation model.

E (kJ/mol)	ΔE (kJ/mol)	% in the population
-2207059.06	0.00	99.94
-2207038.82	20.24	0.028
-2207038.68	20.39	0.027
-2207027.14	31.92	2.56×10^{-4}
-2207027.08	31.98	2.50×10^{-4}
-2207022.11	36.95	3.36×10^{-5}
-2207021.70	37.36	2.84×10^{-5}

Table S3. Energies (E), relative energies (ΔE) in kJ/mol and Boltzmann population distribution at 298 K of conformers of the compound **11*R***, in a range of 50 kJ/mol (12 kcal/mol), calculated with a hybrid DFT (B3LYP) approach with 6-31G* basis set using SM8 ($CHCl_3$) solvation model.

E (kJ/mol)	ΔE (kJ/mol)	% in the population
-2207031.58	0.00	84.42
-2207026.44	5.14	10.63
-2207022.56	9.02	2.22
-2207022.02	9.56	1.78
-2207020.38	11.21	0.92
-2207011.68	19.90	0.03

Table S4. Energies (E), relative energies (ΔE) in kJ/mol and Boltzmann population distribution at 298 K of conformers of the compound **11*S***, in a range of 50 kJ/mol (12 kcal/mol), calculated with a hybrid DFT (B3LYP) approach with 6-31G* basis set using SM8 ($CHCl_3$) solvation model.

E (kJ/mol)	ΔE (kJ/mol)	% in the population
-2207029.90	0.00	62.68
-2207027.67	2.23	25.45
-2207025.27	4.63	9.68
-2207020.40	9.50	1.36
-2207018.62	11.28	0.66
-2207015.21	14.69	0.17
